# Coffee With a Hint of Data: Towards Using Data-Driven Approaches in Personalised Long-Term Interactions

**DOI:** 10.3389/frobt.2021.676814

**Published:** 2021-09-28

**Authors:** Bahar Irfan, Mehdi Hellou, Tony Belpaeme

**Affiliations:** ^1^ Centre for Robotics and Neural Systems, University of Plymouth*,* Plymouth, United Kingdom; ^2^ Polytech Sorbonne, Paris, France; ^3^ IDLab-imec, Ghent University, Ghent, Belgium

**Keywords:** personalisation, task-oriented dialogue, long-term human-robot interaction, few-shot learning, lifelong learning, dataset, data-driven architectures, conversational artificial intelligence

## Abstract

While earlier research in human-robot interaction pre-dominantly uses rule-based architectures for natural language interaction, these approaches are not flexible enough for long-term interactions in the real world due to the large variation in user utterances. In contrast, data-driven approaches map the user input to the agent output directly, hence, provide more flexibility with these variations without requiring any set of rules. However, data-driven approaches are generally applied to single dialogue exchanges with a user and do not build up a memory over long-term conversation with different users, whereas long-term interactions require remembering users and their preferences incrementally and continuously and recalling previous interactions with users to adapt and personalise the interactions, known as the *lifelong learning* problem. In addition, it is desirable to learn user preferences from a few samples of interactions (i.e., *few-shot learning*). These are known to be challenging problems in machine learning, while they are trivial for rule-based approaches, creating a trade-off between flexibility and robustness. Correspondingly, in this work, we present the text-based Barista Datasets generated to evaluate the potential of data-driven approaches in generic and personalised long-term human-robot interactions with simulated real-world problems, such as recognition errors, incorrect recalls and changes to the user preferences. Based on these datasets, we explore the performance and the underlying inaccuracies of the state-of-the-art data-driven dialogue models that are strong baselines in other domains of personalisation in single interactions, namely Supervised Embeddings, Sequence-to-Sequence, End-to-End Memory Network, Key-Value Memory Network, and Generative Profile Memory Network. The experiments show that while data-driven approaches are suitable for generic task-oriented dialogue and real-time interactions, no model performs sufficiently well to be deployed in personalised long-term interactions in the real world, because of their inability to learn and use new identities, and their poor performance in recalling user-related data.

## 1 Introduction

Incrementally learning and recalling aspects about a user to personalise interactions is needed for coherent and lifelike human-robot interactions (HRI) (Lim et al., 2011). For instance, personalised service robots may facilitate feelings of familiarity, trust and rapport with users that encourage them to revisit a shop or restaurant (Kanda et al., 2010; Niemelä et al., 2019). Moreover, personalisation can increase task efficiency and awareness of the situational context of the conversation (Neururer et al., 2018; Kocaballi et al., 2019). In addition, personalisation can facilitate user engagement and responsiveness in long-term HRI after the novelty effect wears off (Dautenhahn, 2004; Bickmore and Picard, 2005; Kanda et al., 2010; Leite et al., 2013; Irfan et al., 2019), and it can overcome negative user experiences (Irfan et al., 2020a).

In order to ensure a natural interaction with robots, in addition to achieving effective communication, the robots need to support natural language interaction (Mavridis, 2015). However, conversations with a robot are challenging, because users may assume multi-modal capabilities based on the various sensors of the robot (e.g., camera, microphones, speakers, tablet) (Goodrich and Schultz, 2007; Rickert et al., 2007), as well as expect the robot to recognise them and recall their previous interactions. In addition, speech may have different accents, grammatical errors and disfluencies, which makes the interaction more challenging. Most solutions in HRI either rely on touch screens or tele-operated robots (e.g., Kanda et al., 2010, Lee et al., 2012, Leite et al., 2017, Glas et al., 2017, Kanda et al., 2010; Lee et al., 2012; Leite et al., 2017; Glas et al., 2017) to bypass these issues, or use rule-based methods in structured transaction-oriented interactions by matching user responses to predefined templates (e.g., Kanda et al., 2007, Churamani et al., 2017, Zheng et al., 2019, Kanda et al., 2007; Churamani et al., 2017; Zheng et al., 2019). However, rule-based approaches are inflexible to the variations in the user responses and are often experienced as time consuming and frustrating (Williams et al., 2018; Bartneck et al., 2019; Irfan et al., 2020a). Moreover, automatic speech recognition errors may arise from various accents, quietly speaking users and pronunciation errors of non-native speakers, which could decrease the robustness of rule-based approaches (Irfan et al., 2020a).

Recent advances in data-driven conversational agents, which rely on extracting and learning the structures and values directly from the training data, allow creating more flexible systems that do not require any feature engineering or domain-specific handcrafted rules (e.g., Sutskever et al., 2014, Graves et al., 2014, Sukhbaatar et al., 2015, Rajendran et al., 2018, Shum et al., 2018, Ram et al., 2018, Roller et al., 2020, Adiwardana et al., 2020, Sutskever et al., 2014; Graves et al., 2014; Sukhbaatar et al., 2015; Rajendran et al., 2018; Shum et al., 2018; Ram et al., 2018; Roller et al., 2020; Adiwardana et al., 2020). However, previous research in data-driven approaches focuses on having a single dialogue exchange with a single user, that is, a memory is not built up over a long-term conversation with different users (Dodge et al., 2016). For conversations with a robot over long-term interactions, the dialogue model would need to learn users and their preferences incrementally, and recall previous interactions with users to adapt and personalise the interactions, which is a *lifelong (or continual) learning* problem. Moreover, the robot should be able to learn new users and their preferences from a few samples of interactions (i.e., *few-shot learning*). While this is a trivial task for a rule-based approach relying on a knowledge-base, lifelong and few-shot learning are challenging problems for data-driven approaches (Parisi et al., 2019; Triantafillou et al., 2017; Madotto et al., 2020) that have not been previously explored for user-specific personalisation in task-oriented long-term interactions. Moreover, there are no publicly available corpora for this task to train or evaluate data-driven architectures.

This work addresses data-driven dialogue models and personalisation in long-term HRI. As context, we use task-oriented interactions between a customer and a robot barista in a coffee shop. In order to evaluate the state-of-the-art data-driven dialogue architectures that were strong baselines in other applications of personalisation (Joshi et al., 2017; Zhang et al., 2018), and create a set of rules for a rule-based dialogue manager for generic and personalised barista robots (Irfan et al., 2020a), two simulated text-based Barista Datasets were created. 1) The Barista Dataset with generic interactions of a customer with a barista, and 2) the Personalised Barista Dataset with personalised long-term interactions, where the barista would recognise the users and learn and recall their preferences. The latter is the first dataset for exploring user-specific personalisation in task-oriented long-term interactions. The Personalised Barista Dataset also contains incorrect user recognition and recall of user preferences, since such circumstances can be experienced in real-world interactions with a robot. The datasets address lifelong and few-shot learning problems through various tasks of increasing difficulties with the presence of out-of-vocabulary entities. This work describes these datasets and explores the potential of data-driven architectures in generic and personalised task-oriented dialogue for long-term interactions.

The Barista Datasets are available online^1^. They are created in the format suitable for ParlAI^2^ (Miller et al., 2017) (i.e., line numbers for each dialogue, and tab-separated customer and bot utterances) platform such that the available data-driven dialogue models on that platform can be used for evaluations, in addition to the goal of contributing to the research community in evaluating their algorithms for personalisation in long-term interactions. The Barista Datasets are explained in detail in Section 2.2.1.

## 2 Materials and Methods

### 2.1 Related Work

While there are available corpora for restaurant bookings (Henderson et al., 2014; Bordes et al., 2017; Joshi et al., 2017) or travel bookings (Hemphill et al., 1990; Bennett and Rudnicky, 2002; El Asri et al., 2017) based on Wizard-of-Oz human-machine interactions (i.e., a robot is tele-operated without knowledge of the user) or simulated datasets (Serban et al., 2018), there was no publicly available corpus on the barista or personalised barista dialogues with customers at the time of conducting this work (January 2019) or the barista robot study (August 2019) (Irfan et al., 2020a). In October 2019, Taskmaster^3^ (Byrne et al., 2019) was released, which contains conversations with a personal digital assistant through Wizard-of-Oz or by “self-dialog” (i.e., crowdsourced workers imagined having a dialogue with a personal digital assistant and wrote the interaction for both sides). Taskmaster contains conversations for ordering drinks at a coffee shop for pick-up at a store (changes to the order only if the drink is not available, and no snack orders), in addition to ordering a pizza, creating auto repair appointments, setting up a ride service, ordering movie tickets, and making restaurant reservations. However, Taskmaster does not contain customer names or personalised subsequent interactions to evaluate personalisation in long-term interactions.

There are only two publicly available datasets that evaluate “personalisation” in task-oriented or open-domain dialogue in English: Persona-Chat (Zhang et al., 2018) and Personalized bAbI dialog (Joshi et al., 2017) datasets. Persona-Chat dataset contains text-based open-domain conversations from crowdsourced workers that were provided sentences determining their *personality* for the dialogue. On the other hand, focusing on the same domain (i.e., task-oriented dialogue) as this paper, Personalized bAbI dialog dataset is a simulated text-based personalised dataset built upon the bAbI dialog (Bordes et al., 2017) dataset for restaurant booking. The dataset focuses on adapting conversation and recommendation styles based on the user’s gender and age, along with restaurant recommendation based on the dietary preferences and favourite food item of the user. However, the Personalized bAbI dialog dataset focuses on personalising the dialogue based on users’ general attributes (gender and age), instead of adapting to each user, which is the focus of this work. Moreover, user attributes are pre-defined at the beginning of each dialogue, instead of obtained from the interaction. Both of these datasets consider only a single user interaction, instead of long-term interactions. Public personalisation datasets available in other languages are: XPersona (Lin et al., 2020) (extension of Persona-Chat in Chinese, French, Indonesian, Italian, Korean, and Japanese) and Pchatbot (Qian et al., 2021) (open-domain dialogue in Chinese with user ID and timestamps). The other datasets that contain personalised task-oriented dialogue, such as dialogues from a coffee ordering service in China (Mo et al., 2017, 2018) (similar to our work)^4^ or persona-based dialogue, such as microblogs with user profile information (in Chinese) (Yang et al., 2017, 2018; Qian et al., 2018; Yang et al., 2021), Reddit dialogues with personas (Mazaré et al., 2018), Twitter conversation corpus with user identity information (Bak and Oh, 2019), and PersonalDialog (in Chinese) for personalised open-domain dialogue based on speaker traits (Zheng et al., 2020a), are not publicly available.

### 2.2 Datasets

#### 2.2.1 Barista Dataset for Generic Task-Oriented Dialogue

The Barista Dataset is designed to model a real-world barista that would: 1) greet the customer and take a drink order, 2) size, and 3) snack, 4) confirm the order, 5) change the order if requested, 6) take the customer’s name, 7) note the pick-up location for the order, and 8) say goodbye. In a typical interaction, a customer can ask for all the order items in one sentence, however, the order steps are separated to reduce the errors in rule-based (e.g., template matching) or data-driven approaches, and to aid speech recognition for spoken dialogue systems, such as a robot.

Similar to the bAbI dialog (Bordes et al., 2017) and the Personalized bAbI dialog (Joshi et al., 2017) datasets, dialogue tasks are identified based on the sequential interactions, as described above. On the other hand, contrary to the bAbI datasets that structure tasks based on application program interface (API) calls or knowledge-base facts, Barista Dataset tasks focus on the different interaction types of dialogues (e.g., ordering a drink, making changes to order) in increasing difficulty of the interaction. The “greetings” of the agent are separated, because using the name obtained during a conversation may decrease the performance for data-driven approaches. Moreover, greetings (e.g., requesting drink order, noting item location) may not occur in real-world barista interactions. Correspondingly, the Barista Dataset tasks are defined as follows:

• Task 1 (B1): *Greetings.* This task evaluates 1) greeting and requesting the drink order, 6) taking the customer’s name, 7) noting the pick-up location of the order, and 8) saying goodbye to the customer. No order is made.
• Task 2 (B2): *Order drink (without greetings).* This task evaluates ordering a drink.
• Task 3 (B3): *Order drink with changes.* This task evaluates ordering a drink and changing the order (up to two changes) during the interaction. The probability of a change is 0.5, sampled from a uniform distribution.
• Task 4 (B4): *Order drink and snack.* This task evaluates ordering a drink and a snack. The probability of ordering a snack is 0.5 (i.e., 50% chance), sampled from a uniform distribution.
• Task 5 (B5): *Order drink and snack with changes.* This task evaluates ordering a drink and a snack (50% chance), and changing the ordered items (up to two changes) during the interaction.
• Task 6 (B6): *Order drink and snack with greetings.* This task is the combination of tasks 1 and 4.
• Task 7 (B7): *Order drink and snack with changes and greetings.* This task is the combination of tasks 1 and 5, and contains interaction types from all tasks.

**FIGURE 1:**
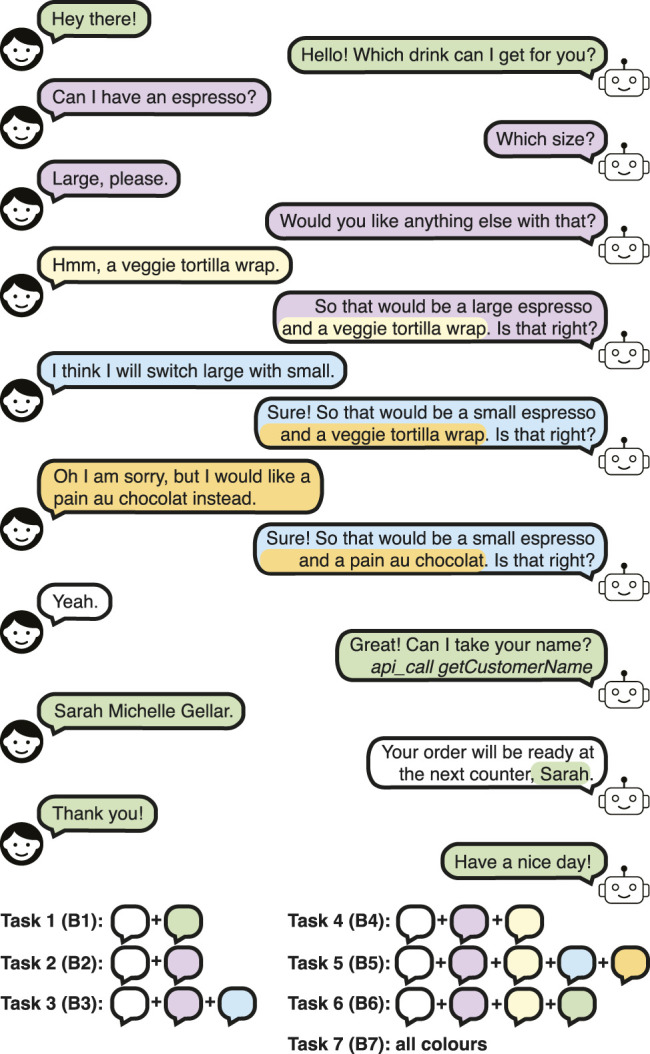
A dialogue example in the Barista Dataset Task 7, showing all the tasks in the dataset.

A dialogue example is given in Figure 1 for task 7. Each interaction type is colour-coded, and the corresponding task number is explained in the legend for clarity. *api_call getCustomerName* refers to an API call to obtain the customer name through another resource (e.g., tablet) or a script, and it is not said to the customer. The number of conversation turns (i.e., user-bot utterance pairs) ranges from 7 (no changes in the order) to 9 (two changes in the order) in the tasks involving orders.

While using a variety of responses for a user utterance can help improve the naturalness of the human-robot interaction, especially within repeated interactions, most data-driven approaches do not take into account that there can be multiple correct next utterances (Rajendran et al., 2018), whereas this is trivial with a rule-based dialogue manager. Correspondingly, we designed a dataset with various multiple bot phrases, which was used for the barista robot (Irfan et al., 2020a), and another dataset with a single phrase for each bot utterance for evaluating data-driven approaches. Nonetheless, in both datasets, customers can use a variety of utterances for each turn.

The Barista Datasets are divided into *training*, *validation* (*development*), *test* and *out-of-vocabulary* (*OOV*) sets. We also refer to the *validation* and *test* set as the *evaluation* sets for brevity. The *training*, *validation* and *test* sets use the same drink, size, and snack types, whereas the *OOV* set contains different drink, size, and snack types that are not part of the other sets. All the order items come from the Starbucks menu^5^, with 20 drink types, 3 sizes, and 20 snacks that the customer can order from in each set. The customer and bot phrases used in the *OOV* set is the same as the other sets.

In order to evaluate the task performance depending on the *training* and *evaluation* set *dataset size*, two sub-datasets are created: 1,000 dialogues (similar to bAbI datasets) and 10,000 dialogues to account for the increased difficulty of the tasks arising from the various names in the dialogues. Supplementary Table S1 in Supplementary Material (SM) 1 presents the *task size* (i.e., the number of customer-bot utterance pairs in the task), the size of the *vocabulary* (i.e., the unique words in a task) for the *test* set, and the *candidate set* (i.e., all bot responses).

The identity of the customer is important for personalising long-term interactions. Moreover, the customer’s (first) name is requested in some coffee shops to separate customer orders and announce when it is ready for pickup. However, in a real-world HRI scenario, the verification of the identity is based only on the customer’s name, thus, using the first name only may cause mixtures of orders, and correspondingly, incorrect online learning of customers and their preferences. Hence, we use the full name of the customer, selected from those in the IMDB-WIKI (Rothe et al., 2015, 2018) celebrity image dataset. The same set of (100) customers (*customer-base A*) appear in *training*, *validation*, and *test* sets, and 100 other customers (*customer-base B*) are in the *OOV* set.

The task difficulty increases when an utterance needs to contain personal information (e.g., customer name) or order details of the customer, as the dialogue architecture should extract this information from the previous exchanges in the dialogue and use it to respond. Hence, we categorise the bot utterances as *personal(ised)* (i.e., containing personal information), *order details* (i.e., containing order item), and *other (remaining)* phrase types, and present the corresponding percentages for each task in the *test* set in Supplementary Table S1 in SM 1, such that we can evaluate the performance of the data-driven approaches in this perspective.

#### 2.2.2 Personalised Barista Dataset for User-Specific Personalisation in Task-Oriented Long-Term Interactions

Recognising “regular” customers and recalling their preferences are important aspects for the long-term deployment of robots in the customer-oriented service domain. While a user can log in to a system with their information (e.g., user ID, email, name) in a text-based interaction (e.g., for chatbots), the customers should be autonomously recognised. In order to integrate this information into the text-based dataset, we use the type of information that can be obtained from user recognition, such as (Irfan et al., 2018): 1) whether the user is known (true/false), 2) the ID of the user (i.e., *0* if the user is new, otherwise, an ID based on the order of the enrolment), and 3) the name of the user. These are sufficient to recall the favourite orders of a user for a rule-based dialogue manager with a knowledge-base, as used in (Irfan et al., 2020a). We extend the Barista Dataset with personalised interactions and user recognition information to create the Personalised Barista Dataset. This dataset contains the interactions from the Barista Dataset for new customers and personalises the interaction for known customers on top of this structure, through recognising customers and suggesting their most common or most recent order in the case of a tie.

As previously mentioned, personalisation in real-world HRI involves incremental and adaptive learning of users, known as lifelong (or continual) learning. However, data-driven approaches may suffer from *catastrophic forgetting*, which refers to the tendency to forget previously learned information upon learning new information (McClelland et al., 1995; McCloskey and Cohen, 1989; Parisi et al., 2019). Moreover, in a real-world interaction, new users will be encountered incrementally, hence the dialogue architecture should be able to respond to new users and learn their preferences without having prior information about them, known as *zero-shot learning*. While this problem is trivial for rule-based dialogue architectures with a knowledge-base, it is a challenging problem for data-driven approaches, as they require a vast amount of data for training (Triantafillou et al., 2017).

In a real-world HRI scenario, especially for long-term deployments, user recognition may not be fully reliable due to noisy data or sensors. In addition, automated speech recognition may not perform well, particularly in a noisy environment or due to various accents, which may cause the dialogue manager to receive incomplete or incorrect information (Irfan et al., 2020a). Moreover, incorrect recalls of user information or knowledge-base entities can cause failures in data-driven approaches (Bordes et al., 2017). Thus, it is important for dialogue managers to account for these errors, and have strategies to recover from failures.

**FIGURE 2:**
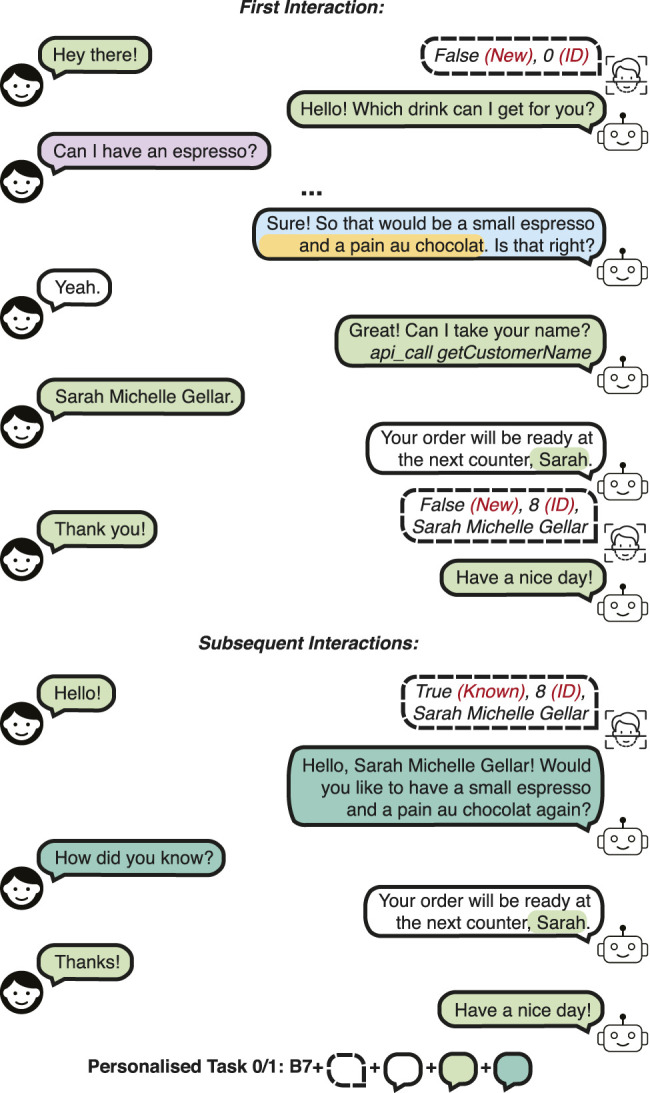
A dialogue example in the Personalised Barista Dataset Task 0 and 1 (confirmed personalised order suggestion).

In order to train and evaluate the data-driven approaches against these challenges, we defined the tasks of the Personalised Barista Dataset as follows:

• Personalised Task 0 (PB0): *Confirmed personalised order suggestion for new customers.* This task aims to evaluate the performance of learning the preferences of a different set of customers than the ones in the training set. The reason we separate this task (and call it Task 0) is that it evaluates zero-shot learning of users. The most common or the most recent drink and snack order of the customer are suggested, and the customer accepts the suggestion. This task assumes perfect recognition and recall, and no changes are made by the customers to their previous preference. An example is given in Figure 2.

The *training* set has 100 users from *customer-base A*, as described in the previous section. However, the *validation* and *test* sets have 100 different users (*customer-base C*). The *OOV* set has 100 users from the *customer-base B* (as in the *OOV* set of the Barista Dataset).

**FIGURE 3:**
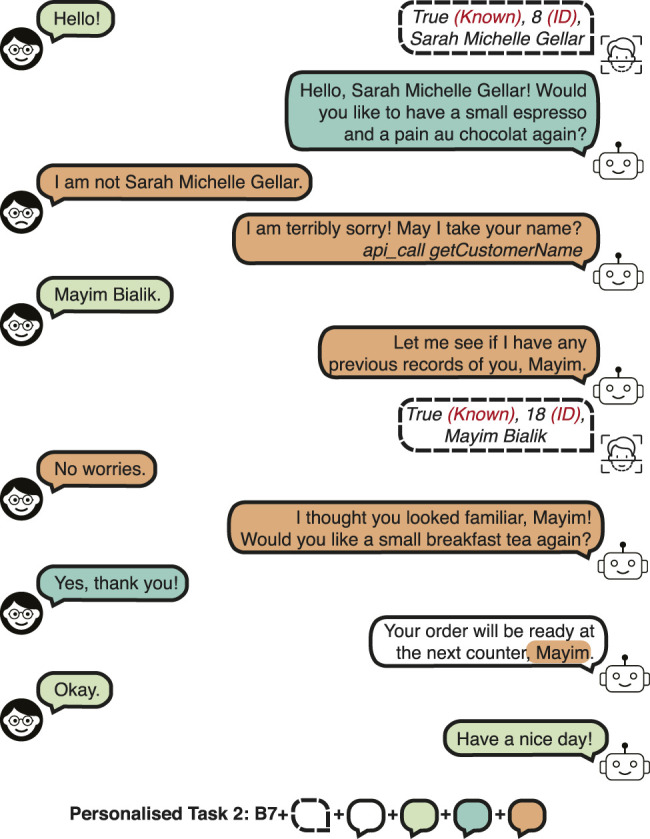
A dialogue example in the Personalised Barista Dataset Task 2 (recognition error).

• Personalised Task 1 (PB1): *Confirmed personalised order suggestion for previous and new customers.* This task requires incrementally learning the preferences of the new ones, as well as remembering the orders of the previous “regular” customers, as in a real-world scenario. Hence, this task contains the same type of dialogue interactions as PB0, but the *validation* and *test* sets also have customers from the *customer-base A*, that is, there are 200 customers in each set from *customer-base A* and *C.* The following tasks build upon this task.
• Personalised Task 2 (PB2): *Recognition error.* This task evaluates recovering from the following type of recognition errors in open world recognition:
  – *Customer is known, but confused with another customer.* A dialogue example is shown in Figure 3.
  – *Customer is known, but not recognised.* The dialogue is conducted as if a new customer is encountered. The error would only be realised during the name request, but no remark or correction is made on the error.
  – *Customer is new, but confused with another customer.* After the customer states that they are not the estimated identity, and the name is requested from the customer, the dialogue is similar to a new customer interaction (e.g., “*It seems to be your first time here! Which drink would you like to have, Rachel?*”).

The detection and identification rate (DIR) of 0.9 and false alarm rate (FAR) of 0.1 are used for the dataset. In other words, 90% of the enrolled customers are correctly recognised, and 10% of the new customers are mistaken for a different customer. A high DIR and a relatively low FAR is used to evaluate whether the data-driven approaches could learn to respond to these errors in the presence of a few erroneous recognitions in the *training* set.

**FIGURE 4:**
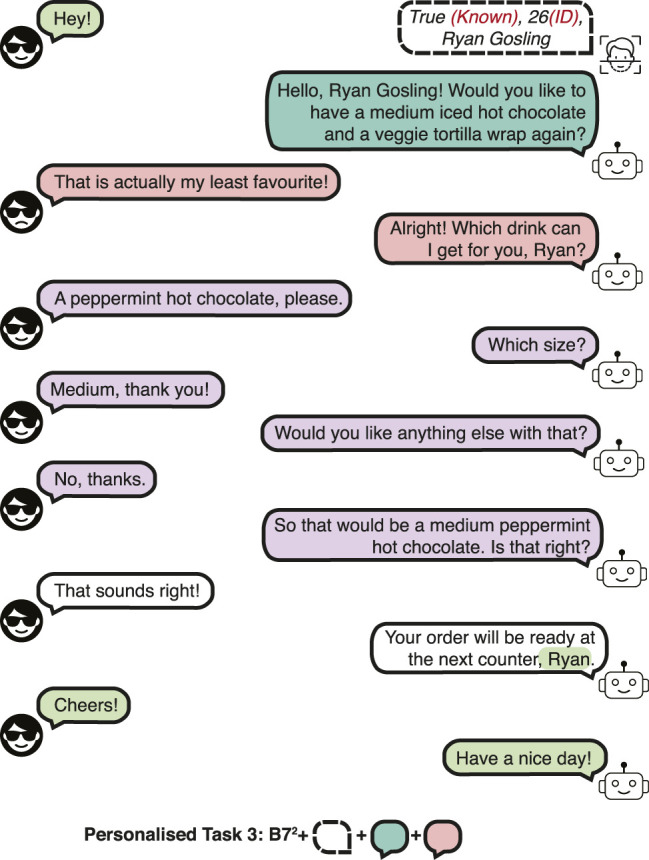
A dialogue example in the Personalised Barista Dataset Task 3 (incorrect recall).

**FIGURE 5:**
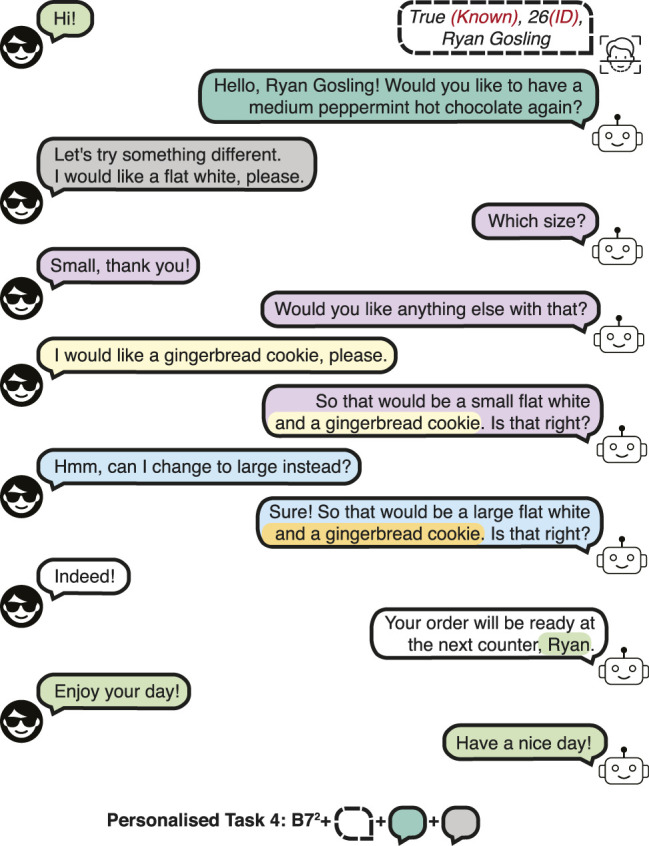
A dialogue example in the Personalised Barista Dataset Task 4 (changes to preference).

• Personalised Task 3 (PB3): *Incorrect recall.* This task evaluates recovering from an incorrect recall of the preferences of the customer. An incorrect memory rate of 0.3 is used, that is 30% of the dialogues contain incorrect recalls of the preferences of known customers. Since the customer preference cannot be correctly suggested, the customer makes a new order, thus, this type of dialogue has phrases from task 7 of the Barista Dataset (B7) for the first and subsequent interactions, denoted as B7^2^. A dialogue example is shown in Figure 4.
• Personalised Task 4 (PB4): *Changes to preference.* This task evaluates the performance when the customer makes a change to their preference, which requires tallying new order items for detecting the most common order items. A change in preference has a probability of 0.5, sampled from a uniform distribution. A dialogue example is presented in Figure 5.
• Personalised Task 5 (PB5): *Recognition error and incorrect recall.* Combines task 2 and 3.
• Personalised Task 6 (PB6): *Recognition error and changes to preference.* Combines task 2 and 4.
• Personalised Task 7 (PB7): *Incorrect recall and changes to preference.* Combines task 3 and 4.
• Personalised Task 8 (PB8): *All tasks.* This task is a combination of tasks 2, 3 and 4. This task evaluates all the scenarios that can occur in a personalised barista interaction.

As previously mentioned, most data-driven approaches require a vast amount of data to train, however, personalised robots deployed in the real-world should learn “on the fly” using only a few interactions, referred to as *few-shot learning*. Thus, in addition to the 1,000 and 10,000 dialogues datasets, we designed the *Second Interaction* dataset, where the *training* set has the initial and the second interactions in task 0 (PB0), and the first, second and third interactions in the remaining tasks to account for learning the previous order of a new user, and learning to count the most common or recent order of a previous user. In the *validation* and *test* sets for PB0, new users will be encountered twice (similar to the *training* set), whereas, for the remaining tasks, the previously known users from the *training* set (from *customer-base A*) will be seen twice and the new users (from *customer-base C*) will only be encountered twice. The number of dialogues per task in the *Second Interaction*, 1,000 and 10,000 dialogue *test* sets are presented in Supplementary Table S2 in SM 1, along with the number of customer-bot utterance pairs (i.e., task size) and the number of unique words in a task (i.e., vocabulary size). The proportions of *personal(ised)* bot utterances (i.e., containing user name or preferences), *order details* (i.e., containing new order or preferences), the *other (remaining)* dialogues, and the phrases belonging to task 7 of the Barista Dataset (B7) are presented for the *test* set in Supplementary Table S2 in SM 1. Note that since both the *personal(ised)* and *order details* phrases can contain user preferences in the Personalised Barista Datasets, the sum of percentages of *personal(ised)*, *order details* and *other* phrases is higher than 100%.

For each task, the orders of the customers are stored in a knowledge-base containing the interaction number, customer identity (i.e., ID number and name), and the final order in the dialogue. A knowledge-base was also used for the rule-based dialogue manager for the personalised barista robot (Irfan et al., 2020a) and evaluating the rule-based approach, however, data-driven approaches do not have access to this information.

#### 2.2.3 Personalised Barista With Preferences Information Dataset

The Personalised Barista Dataset evaluates whether data-driven approaches can learn new customers and track previous conversations to extract their preferences, in addition to using that information to personalise the conversation. Thus, it addresses lifelong learning and user-specific personalisation, which is missing in the currently available datasets. However, the requirement for tracking previous orders and “calculating” the most common order may pose a high level of difficulty for a data-driven approach, which is trivial for a rule-based approach with a knowledge-base. Hence, Personalised Barista with Preferences Information (PBPI) Dataset is created to provide user preference at the beginning of the dialogue alongside the user identity information, to simulate extracting the information from a knowledge-base. For instance, in Figure 1, the information provided will be in the format: *True* (Known), *8* (ID), *Sarah Michelle Gellar* (customer name), *small* (the most common size of the most common drink order), *espresso* (the most common drink order), *pain au chocolat* (the most common snack order). The tasks, the phrases and the corresponding task size, vocabulary size and candidate set sizes of the PBPI Dataset are the same as that of the Personalised Barista Dataset.

### 2.3 Applying Barista Datasets to Human-Robot Interaction

**FIGURE 6:**
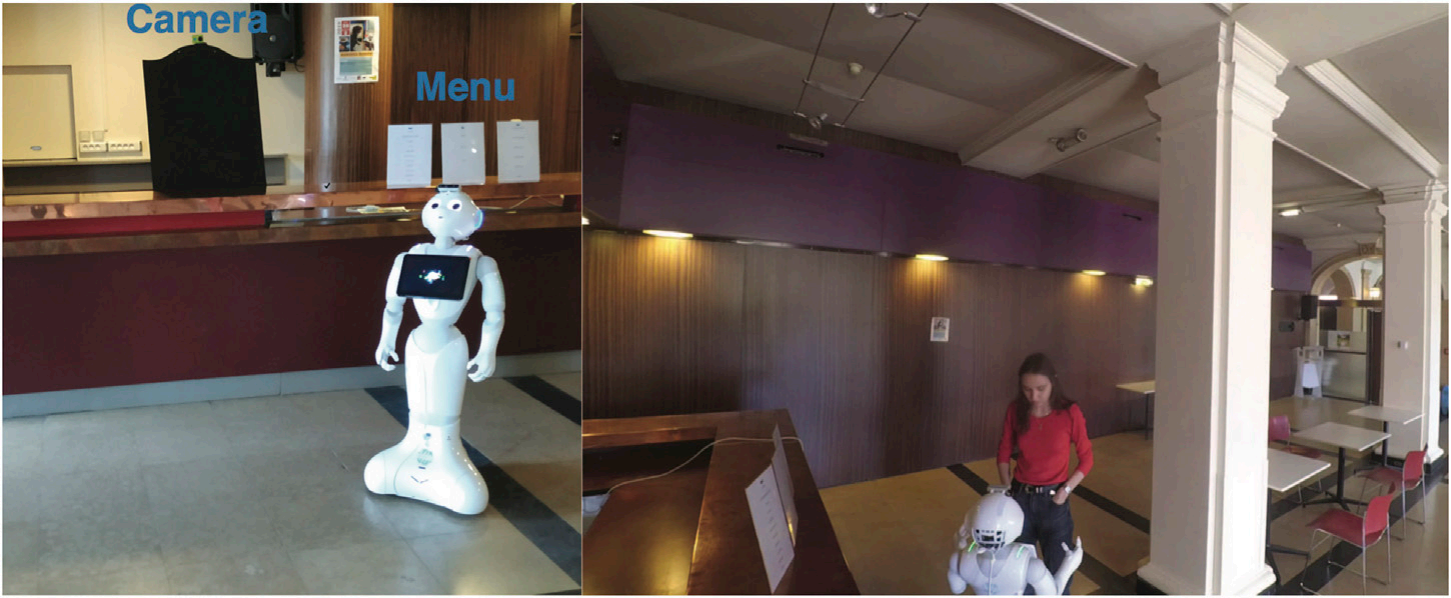
Experiment setup and participant interaction during the barista robot study (Irfan et al., 2020a) at the Cité Internationale Universitaire de Paris (France), with the Adapted Pepper robot.

The Barista Datasets were used in the first real-world study that explores fully autonomous personalisation in dialogue for long-term HRI (Irfan et al., 2020a). Rule-based dialogue managers (through template matching) were built using the datasets to create fully autonomous generic (non-personalised) and personalised barista robots. The robots (*Adapted Pepper* ^6^) were deployed in the coffee bar of an international student campus, Cité Internationale Universitaire de Paris (France), as shown in Figure 6, for 5 days. 18 non-native English speakers (11 males, 7 females) within the age range of 22–47 participated in the study. NAOqi^7^ voice activity detection and Google Cloud Speech-to-Text engine were used for online speech recognition. However, the users’ names were obtained from the robot’s touchscreen interface to avoid misspelling with speech recognition. As previously described, multiple responses (for the same type of query) were used to ensure a less repetitive interaction. In order to ensure a natural level of interaction with mutual understanding (Mavridis, 2015), non-verbal features, such as gaze (through face tracking) and body movements (i.e., animated speech feature of NAOqi), were used. The interaction was personalised by recognising users with Multi-modal Incremental Bayesian Network (Irfan et al., 2018; Irfan et al., 2021), which combines face recognition with soft biometrics (age, gender, height and time of interaction), and a knowledge-base was used to record and recall user preferences.

The results indicated that a rule-based dialogue manager was not robust or flexible enough to the variations in user utterances and for reverting changes in the state of the dialogue. In addition, only 30.2*%* of the utterances were processed by speech recognition, and 55.4*%* of the processed utterances matched correctly to the user utterances (i.e., per-response accuracy), 69.4*%* of the words were correctly recognised (i.e., exact match score) and the BLEU score was 0.49, which are fairly low values considering that the interaction is task-oriented. Correspondingly, incorrect or incomplete phrases were delivered to the rule-based dialogue managers, which further affected their performance, especially for the personalised condition as incorrect users or order items were recorded. In the light of these findings, we decided to divert our attention to data-driven architectures that are gaining popularity in natural language processing, and evaluate their performance on the Barista Datasets for determining the most suitable models for personalisation in long-term HRI before deploying them in a real-world study.

### 2.4 Data-Driven Dialogue Models

Data-driven dialogue models are categorised based on the response generation: *retrieval-based* (ranking or information retrieval) and *generative* models. Retrieval-based models choose a dialogue response from a list of phrases (*candidate set*). While this allows using syntactically correct responses, these models may fail to respond appropriately to novel questions. On the other hand, generative models generate a response word-by-word based on the conversation history (*context*), thus, they can respond with novel responses, however, they are prone to grammatical errors.

Due to the lack of a prior study in user-specific personalisation in task-oriented long-term interactions, this work resorts to the strong baselines from the two publicly available datasets for other applications of personalisation in dialogue, as previously described in Section 2.2: Personalized bAbI dialog (Joshi et al., 2017) and Persona-Chat dataset (Zhang et al., 2018). Correspondingly, Supervised Embeddings (Dodge et al., 2016; Bordes et al., 2017), End-to-End Memory Network (MemN2N) (Sukhbaatar et al., 2015) and Split Memory (Joshi et al., 2017) evaluated on Personalized bAbI dialog, and Sequence-to-Sequence (Sutskever et al., 2014), Generative Profile Memory Network (Zhang et al., 2018), and Key-Value Profile Memory Network (Miller et al., 2016; Zhang et al., 2018), which were the best performing baselines on the Persona-Chat dataset, are evaluated on the Barista Datasets in this work.

Supervised Embeddings is a retrieval-based method that scores the summed bags-of-embeddings of the candidate responses against the summed bags-of-embeddings of the previous conversation to predict the next response. Due to this structure, the order of the words (within the user response and the conversation context) and the repeated words are not preserved, thus, it may not be suitable for dialogue. However, it is selected as a baseline to determine its strong and weak points for user-specific personalisation in comparison to generic dialogue. Seq2Seq is a generative model that uses long short-term memories (LSTM) (Hochreiter and Schmidhuber, 1997; Graves, 2013) as an encoder (read input) and a decoder (produce output). MemN2N is an attention-based model with a long-term memory, where the input (e.g., user query) is weighted with a memory component to find the most relevant previous information for producing an output (e.g., response). Multiple *hops* (i.e., iterating an output with the initial input in multiple layers) enforce the network to increase its attention. The Split Memory architecture combines a MemN2N for conversation context with another MemN2N for the user profile attributes to enforce attention on the user’s profile to improve the accuracy in personalised entities. The Split Memory is equivalent to the MemN2N without the profile information, thus, this method is only evaluated with the Personalised Barista Datasets. Key-Value Memory Network, here referred to as Key-Value for brevity, is an extension of retrieval-based MemN2N by storing facts in key-value structured memory slots. The keys are used to lookup relevant memories to the input, and the corresponding values are read by taking their weighted sum using the assigned probabilities. Generative Profile Memory Network, here referred to as Profile Memory, extends the Seq2Seq model by encoding the profile entries as individual memory representations in a Memory Network. The decoder attends over both the encoded profile entries and the conversation context. Profile Memory is equivalent to the Seq2Seq without the profile information, hence, this method is only evaluated with the Personalised Barista Datasets.

Supervised Embeddings, MemN2N and Split Memory Network are based on the retrieval-based implementations of Joshi et al. (2017) ^8^, and Seq2Seq, Key-Value and Profile Memory Network are based on the implementations of Zhang et al., 2018 in the ParlAI platform. The models, their performance in the literature and the hyperparameters used in this work are described in detail in the Supplementary Material section.

While the *user profile* is identified as the user attributes (e.g., gender, age, favourite food) in the Personalized bAbI dialog and the personality determining sentences (i.e., *persona*) in the Persona-Chat dataset, this work uses the user identity information (i.e., whether the user is enrolled, user’s ID number and name) in the Personalised Barista Dataset, and the user identity information along with the user preferences (i.e., most preferred drink, size and snack) in the Personalised Barista with Preferences Information Dataset.

### 2.5 Research Questions

The following research questions (RQ) are formulated:

• RQ1: *Which architecture is most suitable for generic (non-personalised) task-oriented dialogue?* This question will be explored using the (generic) Barista Dataset.
• RQ2: *Which architecture is most suitable for personalised interactions in task-oriented dialogue?* This will be explored using the Personalised Barista Dataset.
• RQ3: *How much improvement does user preference information provide?* This will be explored using the Personalised Barista with Preferences Information Dataset, in comparison to the performance in the Personalised Barista Dataset.
• RQ4: *What causes inaccuracies in a model?* By examining the performance of the models based on the phrase types, such as *personal(ised)* (i.e., containing user name or preference), *order details*, *other (remaining)* phrases, and generic barista phrases (B7 task), we can infer the underlying reasons for inaccuracies in the models. Moreover, we will examine the dialogue state tracking performance of the models on choosing the correct template corresponding to the dialogue turn.
• RQ5: *What is the effect of out-of-vocabulary (OOV) words, such as new menu items, on the performance?* This will be explored using the *OOV* sets.
• RQ6: *What is the effect of the dataset size? Second Interaction* sets within the Personalised Barista Datasets will provide information on the few-shot learning performance of the models. Moreover, 1,000 and 10,000 dialogue datasets will be compared to evaluate whether increasing the training data size improves performance.
• RQ7: *What is the applicability of the architectures to real-time interaction?* Training and computation time for response generation in the models will be evaluated to understand whether the models can be utilised for real-time interactions.

### 2.6 Experimental Procedure

The experiments relied on the Ghent University IDLab (Belgium) cloud servers and took 6 months (February to August 2020) due to the extensive time required to train Key-Value and Supervised Embeddings models (see Table 1), numerous test cases and the limited amount of resources (e.g., available GPU/CPUs, limited allowance for simultaneous jobs for server usage). The data-driven architectures described in Section 2.4 were trained on the Barista Datasets *training* sets. The models were optimised on the *validation* sets (evaluated after each epoch), and the *test* and *OOV* sets are used to evaluate their performance. The hyperparameters for each method are explained in detail in the Supplementary Material section. These hyperparameters are by no means extensive, and correspond to the hyperparameters from the original implementations (Joshi et al., 2017; Zhang et al., 2018), unless otherwise noted in the text in the Supplementary Material section.

**TABLE 1:** Training and test times of the models for the task 8 of the Personalised Barista Dataset. The test time per example is calculated by dividing the executing time for the task by the number of utterances in each dataset. The MemN2N and Split Memory models have the lowest time complexity.

Dataset	Dataset size	MemN2N	Split memory	Key-value	Profile	Seq2Seq	Supervised
Training time (hours)	300	**0.02**	0.03	2.70	0.86	0.21	3.98
	1,000	**0.08**	0.09	13.79	2.13	0.87	30.58
	10,000	**1.46**	1.65	1,049.87	29.55	6.18	805.28
Test time per example (seconds)	400	**0.0005**	0.001	0.25	0.06	0.15	0.29
	1,000	**0.0006**	**0.0006**	0.39	0.22	0.27	0.41
	10,000	**0.0003**	0.0004	0.68	0.52	0.15	1.22

Key-Value, Generative Profile Memory Network and Sequence-to-Sequence were trained and evaluated using the ParlAI^9^ (Miller et al., 2017) framework with PyTorch (1.1.0) on Python 3.6, while the End-to-End Memory Network (MemN2N), Split Memory and Supervised Embeddings use Tensorflow (1.13.1) on Python 3.6, without an external framework. A Docker^10^ container was created with the code for the modified baselines^11^ and the datasets, and the experiments were run in parallel on (a limited number of) cloud servers for each baseline. Each model was separately trained on each task, that is, the trained model on task 1 is not used for training on task 2.

Each *training*, *validation* and *test* set is randomly divided into batches of dialogue examples, where the *conversation context* (i.e., the conversation history), the *user query* (i.e., the last user response), and the *correct response* (for the bot) are given. All methods have access to the *candidate set* (i.e., set of all bot responses) from all sets during training and test. The model performance is measured by the *per-response accuracy metric* (Bordes et al., 2017; Joshi et al., 2017), which is the percentage of correct matches (i.e., *predicted response* is equal to the *correct response* in text or embedding) within the total number of examples. Both retrieval-based and generative models are evaluated using the per-response accuracy metric, because the correctness of the response determines the success of a real-world interaction for task-oriented dialogue.

Beyond the intrinsic difficulty of each task, *OOV* sets evaluate whether the models could generalise to new entities (i.e., drinks, size, and snacks) unseen in any training dialogue, which embedding methods are not capable of doing (Bordes et al., 2017). Persona-Chat, and (bAbI and) Personalized bAbI dialog papers have a different approach to this evaluation. Persona-Chat evaluations build a vocabulary from the *training*, *validation* and *test* sets leaving out the *OOV* set, and replaces unknown words with a special token. On the other hand, bAbI dialog evaluations add OOV words to the vocabulary during training due to the fixed size vectors used in MemN2N, Split Memory and Supervised Embeddings. In order to remain faithful to reproducing these approaches within a different context, this structure was not changed. Moreover, removing the OOV words from the vocabulary caused erroneous performance measurement in the latter methods. Thus, *OOV* results should be cautiously examined.

## 3 Results

This section presents the findings from the evaluations of the state-of-the-art data-driven dialogue architectures on the Barista Datasets, explored under the research questions formulated in Section 2.5. The best performing methods or the methods that perform within 0.1*%* margin of the best performing method are highlighted in bold for the per-response accuracy metric, similar to (Bordes et al., 2017)^12^. The performance of each method is reported in the order of its average rank in performance within all tasks for the Barista and Personalised Barista Datasets (Section 3.1 and Section 3.2). The remaining analyses focus on the key aspects and implications of the results (Section 3.3–3.7). It is important to note that a rule-based dialogue manager using template matching on the Barista Datasets achieves 100*%* accuracy on the Barista Datasets (Irfan et al., 2020a), because the datasets were created from a set of rules with deterministic bot utterances.

### 3.1 Generic Task-Oriented Dialogue

The performances of the data-driven models on the Barista Dataset in the *test* set on 1,000 dialogues (presented in Table 2) show that Sequence-to-Sequence (Seq2Seq) and End-to-End Memory Network (MemN2N) are suitable for generic task-oriented dialogue.

**TABLE 2:** The *test* set results of the Barista Dataset with 1,000 dialogues. The best performing methods (or methods within 0.1% of best performing) are given in bold for the per-response accuracy metric. The results show that on average and for task 7 (containing all tasks), Seq2Seq is the best performing model, providing near-perfect accuracy.

Task	MemN2N			Key-value	Seq2Seq	Supervised
	Hop1	Hop2	Hop3			
1	**100**	**99.98**	**100**	98.8	99.85	98.72
2	**100**	**99.98**	**99.95**	75.3	**99.92**	76.33
3	97.96	97.73	97.67	65.9	**99.98**	63.59
4	93.33	95.85	98.45	65.7	**99.95**	85.25
5	91.97	79.92	94.85	60.46	**98.97**	69.54
6	96.66	98.7	99.69	78.27	**100**	89.64
7	94.78	96.29	95.99	70.98	**99.85**	80.25

**Sequence-to-Sequence: *Best model*** Seq2Seq model performs best for generic task-oriented dialogue within all models, except in the first task where it is a close second. Given that Seq2Seq is a generative model which forms sentences word-by-word, these remarkable results show that the model could learn both the grammar and the correct responses well, in contrast to the retrieval-based methods which only need to learn the correct responses. The model also achieves a near-perfect accuracy, showing that it is suitable for generic task-oriented dialogue.

**End-to-End Memory Network:** While MemN2N achieves 100% accuracy in the greetings (B1) and ordering a drink without greetings (B2) tasks, the introduction of changes (B3) and an additional order item (B4) worsened its performance. Even though using 2 hops provides the best of MemN2N in B7 (that contains interaction types from all tasks), this model performed poorly in B5. On average, using 3 hops performs the best, which suggests the importance of focusing the attention. The overall good performance of the model suggests that it is also suitable for generic task-oriented dialogue.

**Supervised Embeddings:** While the model was shown to be a strong baseline in the literature, the results show that it does not perform as favourable as the other models, despite its good performance in greetings. Moreover, as previously mentioned, the Supervised Embeddings model does not preserve the order of the words within the sentence or the time order of the conversation context, because the words in the user and bot utterances and the conversation context are embedded according to their order in the vocabulary, which resulted in a poor performance in changes in the order (B3) task. Correspondingly, the implementation of Joshi et al., 2017 is not suitable for task-oriented dialogue. An implementation that uses an embedding to preserve the word order in the utterances and the context of the conversation would be more suitable and may provide different results.

**Key-Value Profile Memory Network:** While Key-Value was the best performing model in the open-domain Persona-Chat dataset, the poor performance on the Barista Dataset suggest that it is not suitable for generic task-oriented dialogue. The initial good performance of models in the greetings (B1) task may be attributed to its *chit-chat* capabilities in open-domain dialogue.

### 3.2 Personalised Task-Oriented Dialogue

The performances of the data-driven models on the Personalised Barista Dataset in the *test* set on 1,000 dialogues (presented in Table 3) show that none of the models performed sufficiently well (i.e., above 90 accuracy%) to be deployed in real-world personalised long-term interactions. The performance drops considerably below the level in the generic task-oriented dialogue, indicating that personalisation in long-term interactions is a challenging problem for the state-of-the-art data-driven approaches. The drastic loss of performance can be explained by the *catastrophic forgetting* problem in lifelong learning. This is particularly apparent from the substantial drop of accuracy in the *test* set which contains completely different users than those in the *training* set (PB0) when compared with the addition of users from the *training* set (PB1), except for Key-Value, which surprisingly performs equally well in tasks 0 and 1. The results also indicate that the presence of user recognition errors (PB2) and incorrect recalls (PB3) decreased the performance of the models, which may arise from the increased number of turns and the misinformation about the customer or their order, however, these tasks are necessary to train the models to handle such situations in the real world, especially for fully autonomous robots. Below the performance of each model is analysed in detail.

**TABLE 3:** The *test* set results of the Personalised Barista Dataset with 1,000 dialogues. The best performing methods (or methods within 0.1% of best performing) are given in bold for the per-response accuracy metric. The results show that on average and for task 8 (containing all tasks), MemN2N is the best performing model.

Task	MemN2N			Split memory			Key-value	Profile	Seq2Seq	Supervised
	Hop1	Hop2	Hop3	Hop1	Hop2	Hop3				
0	42.52	43.01	43.27	44.97	44.89	44.63	**61.91**	40.91	40.28	55.85
1	70.42	70.97	70.82	**71.83**	71.34	71.05	61.97	69.73	50.21	67.03
2	69.35	69.32	69.77	**70.25**	70.06	69.93	55.28	69.32	41.45	63.65
3	**68**	67.22	67.51	66.24	67.53	66.69	47.65	61.42	46.24	62.06
4	72.71	**76.58**	75.67	72.44	74.42	74.81	47.31	63.86	64.33	61.16
5	62.83	63.82	**65.37**	63.45	62.83	63.8	43.2	59.73	45.13	57.61
6	70.48	**74.71**	73.85	69.98	72.99	72.88	44.38	62.22	54.78	57.12
7	70.42	**72.77**	72.04	68.26	70.2	63.9	43.74	58.18	68.43	60.26
8	68.8	**71.81**	70.01	64.93	66.95	66.95	40.09	57.79	60.58	56.17

**End-to-End Memory Network: *Best model*** On average (in 6 out of 9 tasks) and in task 8, which contains all non-generic and personalised dialogues, MemN2N performs best in all models, especially using 2 hops. However, in task PB0, the performance is poor and below that of the Key-Value and Split Memory, indicating that the model is not suitable for interactions with new users. Moreover, it performs slightly worse than Split Memory for PB1 and recognition errors (PB2). Nonetheless, it is more competent in handling incorrect recalls (PB3) and changes to preference (PB4) than all other models, which may indicate that it is capable of changing an incorrect order and tallying previous orders to suggest the most preferred order.

**Split Memory Network:** Split Memory is the second-best model for personalised task-oriented dialogue, however, our initial expectations based on the findings of Joshi et al., 2017 were that it would outperform MemN2N, because it pays separate attention to the user identity information (as presented in Supplementary Table S17 in SM 6), which may be the underlying reason of its superior performance to MemN2N in tasks PB0, PB1 and PB2 (recognition error). However, for overcoming incorrect recalls (PB3) and making changes to preference and tallying (PB4), the model performs slightly worse than MemN2N. This result may be due to its inferior performance in issuing API calls (e.g., suggesting the user preference) and updating the response according to changes in the user requests, as reported in (Joshi et al., 2017), confirming that a simpler MemN2N model is more suitable for tasks which do not require compositional reasoning over various entries in the memory.

**Supervised Embeddings:** Within a close competition with the Generative Profile Memory Network, the Supervised Embeddings is the third-best model on average. In contrast with the findings of Joshi et al., 2017, where the model performed very poorly (12%) in updating API calls, the model performance was not considerably affected when handling changes to the preference (PB4) or the order (in the Barista tasks B3 and B5).

**Generative Profile Memory Network:** Having a separate profile memory allows focusing on the user identity information, thus, the model performs better than Seq2Seq in tasks focusing on such information. However, it performs worse in some of the tasks that involve changes to the preference (PB4, PB7, PB8). The most prominent reason could be using the model predictions instead of the correct labels in the *validation* and *test* sets, which may have decreased its performance in tracking the dialogue state or the order items in the dialogue. However, as described in the Supplementary Material section, using correct labels in these sets, surprisingly decreases the performance of the model.

**Sequence-to-Sequence:** Despite achieving near-perfect accuracy in generic task-oriented dialogue (on the Barista Datasets), the Seq2Seq model does not perform well in personalising the dialogue.

**Key-Value Profile Memory Network:** Within open-domain dialogue, Key-Value was found to considerably outperform (30–50% accuracy) both Profile Memory and Seq2Seq (8–10% accuracy) as well as the other retrieval-based models (Zhang et al., 2018), however, our evaluations show the contrary in task-oriented dialogue. Nonetheless, the results suggest that the model is indifferent to the customer database (i.e., performing almost equally in PB0 and PB1), which is a highly important aspect, for instance, for deploying the robot with new users or in different locations of the same coffee shops. However, this model is not able to handle neither the inaccuracies of real-world dialogue (PB2 and PB3), nor the changes in customer preferences.

### 3.3 User Preferences Information

Rule-based approaches relying on a knowledge-base have the advantage of knowing the preferences of the user prior to the conversation. Data-driven architectures would need to recall the previous orders of the user and tally the orders to obtain the most common preference, which is a very challenging problem. Thus, user preference information was provided in the Personalised Barista with Preferences Information Dataset, as described in Section 2.2.3, alongside the user identity information, similar to the Personalized bAbI dialog dataset (Joshi et al., 2017). The resulting performances of the data-driven models on the Personalised Barista with Preferences Information Dataset in the *test* set on 1,000 dialogues are presented in Table 4.

**TABLE 4:** The *test* set results of the Personalised Barista with Preferences Information Dataset with 1,000 dialogues. The best performing methods (or methods within 0.1% of best performing) are given in bold for the per-response accuracy metric. The results show that on average MemN2N is the best performing model, however, Seq2Seq performs best for the task 8 (containing all tasks).

Task	MemN2N			Split memory			Key-value	Profile	Seq2Seq	Supervised
	Hop1	Hop2	Hop3	Hop1	Hop2	Hop3				
0	42.9	41.75	43.01	43.39	44.31	43.04	**62.37**	41.37	40.33	53.05
1	70.71	70.42	70.19	70.59	70.39	**70.91**	62.28	69.13	42.62	69.56
2	68.84	68.92	68.82	**69.32**	69.16	**69.29**	54.54	67.65	40.79	65.75
3	**70.3**	**70.26**	70.05	68.44	68.06	69.01	49.39	62.67	66.73	65.27
4	73.6	76.15	75.92	74.17	75.96	**76.31**	50.9	63.68	68.92	67.15
5	65.93	**66.2**	**66.13**	64.47	65.23	64.76	45.35	60.82	62.48	61.86
6	71.94	74.82	**75.15**	71.14	74.3	74.21	45.86	62.31	52.51	61.96
7	73.66	74.34	73.88	70.65	72.19	72.96	45.72	59.12	**75.21**	63.47
8	73.19	72.98	73.3	68.75	71.65	69.73	42.95	58.96	**76.16**	60.66

Our initial expectations were that preferences information would increase the accuracy of the models, especially for tasks focusing on learning and recalling the user preference (PB0, PB1, PB4). However, when Table 3 and Table 4 are analysed comparatively, this information seems to have a varying effect depending on the task and model. Nonetheless, it improved the accuracy in all models for task PBPI8 offering up to an increase of 15.58% (for Seq2Seq). Similar to the previous results on the Personalised Barista Dataset, MemN2N performs the best on average, however, the Seq2Seq model performs best in task PBPI8. Despite the increase in accuracy, the overall accuracy remains considerably below that of the rule-based dialogue manager (100%) and below a sufficient level for deployment (90%), consequently, no model is adequate for personalised long-term interactions in the real world.

### 3.4 Reasons for Inaccuracies

In the previous sections, the performances of the data-driven approaches were compared on a task basis. However, the overall accuracy in a task does not provide sufficient information on the underlying reasons for the inaccuracies in the models. Thus, logs were recorded during the evaluation in the *test* sets for categorising errors according to the phrase types previously presented in Supplementary Table S1 and Supplementary Table S2 (in SM 1), namely, in terms of *personal(ised)* (i.e., containing customer name or preferences), *order details* (i.e., containing order item), *other* (remaining) phrases and the phrases that appear in the Barista Task 7 (B7). Moreover, the dialogue state tracking errors were analysed based on whether the model responded with the correct template (excluding customer name or the order item) for the conversation turn. Table 5 presents the error percentages based on these categories for the tasks that contain all the tasks within the Barista (B7), Personalised Barista (PB8) and Personalised Barista with Preferences Information (PBPI8) Datasets, in addition to the personalisation tasks in which the customer preferences are recalled and suggested, and the customers confirm the suggestion (PB0 and PB1). These latter tasks show whether the models can learn and use customer names and preferences for new customers (PB0) and additionally for previous customers (PB1).

**TABLE 5:** Percentage of errors in dialogue state tracking (DST), *personal(ised)*, *order details*, other and Barista Task 7 (B7) phrase types for 1,000 dialogue *test* sets. The best performing methods (or methods within 0.1%) are given in bold for the error in per-response accuracy, and the error percentage within the phrase type is given in parentheses.

Task	Error type	MemN2N			Split memory			Key-value	Profile	Seq2Seq	Supervised
		Hop1	Hop2	Hop3	Hop1	Hop2	Hop3				
B7	DST	0.54	0.35	0.18	-	-	-	1.89	-	**0**	0.78
	Personal	0.36 (2.80)	0.15 (1.20)	0.14 (1.10)	-	-	-	**0.00** (0.00)	-	**0.00** (0.00)	3.87 (30.09)
	Order	4.84 (21.13)	3.48 (15.19)	3.87 (16.87)	-	-	-	20.97 (91.48)	-	**0.15** (0.67)	15.03 (65.58)
	Other	**0.01** (0.02)	**0.08** (0.12)	**0.00** (0.00)	-	-	-	8.05 (12.54)	-	**0.00** (0.00)	0.85 (1.32)
PB0	DST	21	2.13	0.49	22.62	21.78	3.03	28.44	**0**	0.29	1.73
	Personal	53.36 (97.47)	52.58 (96.05)	52.52 (95.95)	50.79 (92.79)	50.16 (91.63)	50.99 (93.16)	**29.13** (53.21)	54.22 (99.05)	54.74 (100.00)	52.95 (96.74)
	Order	**29.85** (96.72)	30.11 (97.56)	**29.90** (96.91)	30.14 (97.65)	30.74 (99.61)	30.14 (97.65)	30.74 (99.61)	30.80 (99.80)	30.86 (99.99)	30.25 (98.03)
	Other	0.20 (0.50)	0.23 (0.57)	0.23 (0.57)	**0.03** (0.07)	0.14 (0.36)	0.17 (0.43)	4.06 (10.07)	**0.00** (0.00)	**0.06** (0.14)	0.86 (2.14)
	B7	**4.12** (5.56)	4.41 (5.95)	**4.21** (5.68)	4.24 (5.72)	4.96 (6.69)	4.38 (5.91)	8.96 (12.10)	4.87 (6.57)	4.98 (6.73)	5.19 (7.00)
PB1	DST	8.97	4.36	0.66	11.84	8.46	0.97	33.08	**0**	0.29	1.63
	Personal	24.79 (45.52)	24.85 (45.63)	24.33 (44.68)	**23.65** (43.42)	24.08 (44.21)	24.62 (45.21)	28.52 (52.37)	24.85 (45.63)	44.22 (81.21)	33.99 (62.42)
	Order	16.19 (51.89)	**15.59** (49.96)	16.05 (51.43)	15.96 (51.15)	15.71 (50.33)	**15.65** (50.14)	30.84 (98.81)	17.02 (54.55)	29.75 (95.32)	24.13 (77.32)
	Other	**0.06** (0.14)	0.14 (0.36)	0.26 (0.64)	**0.03** (0.07)	0.34 (0.86)	0.14 (0.36)	4.21 (10.50)	**0.00** (0.00)	0.14 (0.36)	4.53 (11.28)
	B7	4.79 (6.45)	**4.18** (5.64)	4.84 (6.53)	4.53 (6.10)	4.59 (6.18)	4.33 (5.83)	9.52 (12.82)	5.42 (7.30)	5.56 (7.49)	9.37 (12.63)
PB8	DST	2.81	2.87	4.89	2.57	3.45	1.96	49.67	2.09	**0.29**	9.88
	Personal	**21.24** (55.93)	21.92 (57.70)	22.20 (58.46)	22.11 (58.21)	23.31 (61.38)	23.46 (61.76)	22.33 (58.80)	23.63 (62.22)	25.43 (66.95)	31.53 (83.01)
	Order	20.39 (57.38)	**16.89** (47.54)	18.47 (51.96)	23.68 (66.63)	20.79 (58.50)	20.76 (58.41)	34.11 (95.97)	30.07 (84.60)	29.78 (83.78)	33.72 (94.89)
	Other	0.71 (1.66)	0.56 (1.32)	0.66 (1.55)	0.66 (1.55)	0.58 (1.36)	0.51 (1.21)	18.02 (42.45)	0.71 (1.66)	**0.03** (0.08)	2.68 (6.31)
	B7	9.67 (12.64)	**6.27** (8.20)	7.60 (9.94)	12.87 (16.81)	9.74 (12.72)	9.47 (12.37)	36.05 (47.10)	18.56 (24.25)	14.04 (18.34)	20.47 (26.75)
PBPI8	DST	4.17	2.68	2.89	3.1	2.79	2.39	55.54	**0.34**	0.75	4.2
	Personal	27.56 (72.57)	21.34 (56.18)	21.76 (57.28)	20.55 (54.11)	21.15 (55.68)	21.08 (55.51)	22.00 (57.91)	21.72 (57.20)	**18.76** (49.38)	27.24 (71.73)
	Order	18.02 (50.70)	13.91 (39.14)	**13.27** (37.33)	19.17 (53.95)	16.24 (45.68)	17.92 (50.42)	21.05 (59.23)	29.36 (82.61)	13.52 (38.06)	27.77 (78.14)
	Other	0.85 (2.00)	0.47 (1.10)	0.48 (1.13)	0.48 (1.13)	0.24 (0.57)	0.48 (1.13)	15.48 (36.48)	**0.05** (0.11)	**0.05** (0.11)	2.89 (6.80)
	B7	6.32 (8.26)	5.58 (7.29)	**4.76** (6.23)	10.62 (13.88)	7.19 (9.39)	9.11 (11.91)	33.53 (43.81)	19.30 (25.22)	5.12 (6.69)	19.43 (25.38)

The percentage of errors corresponds to the number of erroneous bot responses corresponding to the category divided by the task size (number of user-bot utterance pairs), that is, it corresponds to the percentage of the error in the overall task performance, which can help identify the most common errors in the overall performance and facilitate equal comparison between the models. The percentage of errors within the parentheses represent the percentage of the error within the respective phrase types, calculated by the number of errors within the phrase type divided by the total number of user-bot utterance pairs in the phrase type. For the Barista Dataset, the sum of errors in *personal(ised)*, *order details* and *other* phrases (i.e., phrases without customer name or order item) equal the total error in the per-response accuracy. In contrast, for the Personalised Barista Datasets, *personal(ised)* phrases also include order items (i.e., for suggesting customers their most preferred order), and B7 phrases also include order details (i.e., order confirmation) and *personal(ised)* phrases (i.e., referring to the customer name for noting the pick-up location). Thus, the total error of phrase types is higher than the overall error. However, analysing each phrase type separately is important to evaluate the errors from different perspectives. Moreover, while the percentage of error within the total utterances may seem low, it may correspond to a high error within the phrase type (given in parentheses). For instance, MemN2N with 1-hop has 29.85% error due to order phrases in the PB0 task, but this makes up 96.72% of all the *order details* phrases, which means that most of the phrases containing an order are wrong. The key points derived from the detailed analysis of the logs based on these categories will be presented in this section.

**New customer names cannot be learned:** All models except Key-Value can only use names that occur in the *training* set, showing that they are not suitable for incremental learning of new names. This is the underlying reason for the poor performance in the *personal(ised)* and *order details* utterances in PB0 in comparison to PB1 or PB8. Similarly, for this reason, having a separate memory for user profile information (i.e., Split Memory, Key-Value, or Profile Memory) or using preferences information (e.g., in PBPI8) does not markedly improve the performance. It should be noted that each model has in its vocabulary the new customer names from *training*, *validation* and *test* sets, as well as candidates from all sets. The *out-of-vocabulary* definition of Bordes et al., 2017; Joshi et al., 2017 corresponds to the previously unseen words (e.g., restaurant names, cuisines) in the *training* set, and those studies showed that the performance decreases substantially in this case^13^. However, those studies did not investigate the underlying reason for these errors.

**Size of the conversation context affects the performance:** Models are more prone to errors in longer conversation context, causing confusions of order items (Supplementary Figure S1 and Supplementary Figure S2 in SM 6) or errors in dialogue state tracking, thus, increasing the percentage of B7 errors, especially in the presence of incorrect recalls or user recognition errors, which increases the number of turns in the dialogue. Also, the entities (e.g., order item or drink size) that did not occur in the conversation context was found to be occasionally used incorrectly in responses within all of the models (Supplementary Figure S3 in SM 6), especially during changes or longer conversation context. Table 5 shows that MemN2N performs best in recalling *order details* in the conversation for personalised task-oriented dialogue, whereas, Seq2Seq is the best in generic task-oriented dialogue.

**User recognition errors and incorrect recalls are frequent in all personalised tasks:** Customers are confused as a new customer or confused with a known customer (even for those in the *training* set), and their preferences are incorrectly recalled, including the models that have a separate memory for user profile information (i.e., Split Memory, Key-Value and Profile Memory). This finding shows the importance of training the models to appropriately react in the presence of these errors in real-world interactions.

**Generative models learn sentence grammar and structure well:** Low dialogue state tracking errors in all tasks shows that despite generating sentences word-by-word, Seq2Seq and Profile Memory performs very well in learning the correct grammar and template for responding to users, with rare errors for missing words in the response (Supplementary Figure S4 in SM 6) or mixing words from different phrases.

**Generative models are better in dialogue state tracking:** Within all tasks in all datasets, Seq2Seq or Profile Memory perform best in dialogue state tracking.

**Time order within the conversation context is important:** Supervised Embeddings cannot track and correctly respond to the changes in the preference, user recognition errors and incorrect recalls, because the bag-of-words embeddings do not preserve the time order of the sentence and the conversation context.

**Key-Value is not suitable for task-oriented dialogue:** Key-Value performs poorly in dialogue state tracking, and the most prominent reason is that it repeats the previous bot utterance. It was found to be the only model that can use the correct customer name, however, it does so frequently within the wrong context. For example, at the first turn in the dialogue, the model responds with *“Alright! Which drink can I get for you, Lena?”*, which is the phrase for the incorrect recall, instead of suggesting the user preference. Moreover, due to using only the last bot-user response pair in the context instead of the full dialogue (which was found to perform better for the model, as stated in Supplementary Material), the performance in the order confirmation is poor because the drink and size of the order are missing from the conversation context. On the other hand, using the bot-user response pair should have improved the performance for changes in the user preferences, since the previous order confirmation and the user change is both available in the context, however, this also does not appear to be the case. Overall, in contrast to its good performance in open-domain dialogue (Zhang et al., 2018), the performance of the model is very low in all Barista Datasets, showing that this model is not suitable for task-oriented dialogue.

### 3.5 Out-Of-Vocabulary

The previous section showed that customer names that are available in the *training* set cannot be used in the responses by most of the models for the *test* sets, as evident by a drastic drop in performance for the models between PB0 and PB1. This section examines the performance of the models on the out-of-vocabulary (OOV) words that correspond to new customer names and order items (i.e., drinks, sizes and snacks) which are not seen in the *training* set. In other words, none of the tasks for the *OOV* sets contains customers or order items from the *training* set.

As previously noted, the definition of out-of-vocabulary in the work of Bordes et al., 2017 and Joshi et al., 2017 differs from that of Zhang et al., 2018: the former work adds the new entities to the vocabulary of the model, whereas the latter does not. In addition, removing the OOV words from the vocabulary for the methods implemented by Joshi et al., 2017 (i.e., MemN2N, Split Memory, Supervised Embeddings) resulted in erroneous accuracy metrics. Thus, this work follows the definition of the original work for each model (i.e., including OOV words into the vocabulary of MemN2N, Split Memory, Supervised Embeddings models, and not including them for the other methods) to have a fair comparison with the reported performance in the original work.

Table 6 presents the *OOV* set results of the Barista Dataset. The remaining *OOV* set results for the 1,000, 10,000 dialogues and Second Interaction datasets and the error percentages for the *OOV* sets are presented in the Supplementary Material section for reasons of perspicuity. Note that the datasets contain recognition errors (PB2) where the *OOV* set customer may be confused with a *training* set customer, so a few *training* set customer names and order items (i.e., preferences) may appear in the *OOV* sets for the Personalised Barista Datasets. This might explain why the *order details* errors in the PB8 and PBPI8 tasks is less than that of B7, PB0 and PB1. Similar to Section 3.4, the key points derived from the detailed analysis of the logs are presented in this section.

**TABLE 6:** The *out-of-vocabulary (OOV)* set results of the Barista Dataset with 1,000 dialogues. The best performing methods (or methods within 0.1% of best performing) are given in bold for the per-response accuracy metric. The results show that on average and for task 7 (containing all tasks), Seq2Seq is the best performing model, similar to the *test* set.

Task	MemN2N			Key-value	Seq2Seq	Supervised
	Hop1	Hop2	Hop3			
1	**79.9**	78.62	76	75.15	76.85	77.45
2	74.05	73.8	70	18.07	**75**	74.28
3	55.07	54.53	58.34	9.01	**62.87**	60.96
4	67.8	65.58	65.7	11.07	**75**	74.58
5	60.07	50.32	60.26	6.7	**63.59**	57.72
6	62.31	59.63	60.03	34.39	71.93	**72.64**
7	55.42	61.54	60.01	32.83	**64.96**	61.67

**Out-of-vocabulary entities decrease the accuracy drastically:** The performance of all the models in all *OOV* sets show that regardless of whether the OOV words are included in the vocabulary or not, all methods have a drastic drop in performance. Comparing the performance of the models on the Barista Dataset *OOV* set (Table 6) with the performance on the *test* sets (Table 2), show that most models lost 20-40% of accuracy. Seq2Seq model performs best in all the models for the Barista Dataset containing OOV entities, however, the Supervised Embeddings model performs best overall in the Personalised Barista Datasets.

**All models perform poorly for new customer names or new orders:** Comparatively analysing the percentage of errors within the phrase types (i.e., the error percentages are given in parentheses) within the *test* (Table 5) and *OOV* (Supplementary Material) sets show that the errors have substantially increased in all models in *personal(ised)* and *order details* phrases (0 to 11.19% per-response accuracy) in the Personalised Barista Datasets, because of the new entities. This finding confirms the previous statement that models cannot learn new names or entities that were not previously seen in the *training* set. The only correct *personal(ised)* phrases in all models except Key-Value correspond to using the phrase “Your order will be ready at the next counter” with customers that have their first name same as a customer in the *training* set.

**Out-of-vocabulary entities increase dialogue state tracking errors:** The dialogue state tracking errors increased in all models because the models confused known customers in the *OOV* set with new customers as the customers in the *OOV* set could not be learned.

**Key-Value can learn new entities, but performs worst in the models due to dialogue state tracking errors:** Despite being able to use OOV entities (i.e., names or orders) even without having these words in the vocabulary, Key-Value performed extremely poorly in *OOV* sets, worse than the other models. The most prominent underlying reason is the higher number of dialogue state tracking errors, especially due to its tendency to repeat the previous bot utterance, as previously noted. Another reason could be due to the increased number of available items, which may have increased incorrect recalls (Supplementary Figure S5 in SM 6), and confusing item names in the dialogue.

**End-to-End Memory Network can learn new order items, but fails to use them:** While MemN2N was not able to use any new customer names, it was able to use the OOV order items on rare occasions. This indicates that the model *can* learn new entities, in contrast to our initial conclusion, however, it *does not*, in general.

### 3.6 Dataset Size

In general, the accuracy of machine learning approaches tends to improve with more data, since the correlations between correct labels and the queries can be learned better with increasing samples (Kottur et al., 2017). On the other hand, learning the patterns in the inputs and outputs from a few samples of data (i.e., few-shot learning) is a challenging problem (Triantafillou et al., 2017). When combined with lifelong learning and out-of-vocabulary words, the intrinsic difficulty of each task becomes very high, thus, the accuracy of the models can drop substantially. Correspondingly, this section evaluates the effects of the dataset size on the per-response accuracy of the models using the Second Interaction dataset that has only 2–3 dialogues per customer in the *training* set, and the 10,000 dialogues datasets with 100 dialogues per customer. Note that directly comparing the performances between Second Interaction, 1,000 dialogues and 10,000 dialogues may result in incorrect conclusions due to the differing percentage of *personal(ised)* and *order details* phrases between the datasets. Thus, the percentage of errors within the phrase types across datasets (based on Table 5, Supplementary Table S13 in SM 4, Supplementary Table S15 in SM 5) are compared, along with the performance of the models within each dataset based on the per-response accuracy.

#### 3.6.1 Second Interaction

Table 7 shows the few-shot learning performance of the models on the Personalised Barista Dataset using the Second Interaction set. The performance of the models on the Personalised Barista with Preferences Information Dataset and the error analysis based on phrase types are presented in the Supplementary Material section.

**TABLE 7:** The *test* set results of the Personalised Barista Dataset with Second Interaction set (few-shot learning). The best performing methods (or methods within 0.1% of best performing) are given in bold for the per-response accuracy metric. The results show that on average and for task 8 (containing all tasks), Seq2Seq is the best performing model.

Task	MemN2N			Split memory			Key-value	Profile	Seq2Seq	Supervised
	Hop1	Hop2	Hop3	Hop1	Hop2	Hop3				
0	59.3	58.55	58.83	59.02	**59.49**	57.99	55.17	56.02	56.95	43.99
1	74.19	74.27	73.34	73.27	**75.27**	73.81	54.62	71.26	62.56	61.52
2	70.06	68.76	68.11	68.4	**70.56**	70.2	46.97	70.13	60.46	55.99
3	62.41	62.85	62.66	63.48	62.85	62.28	45.51	**64.81**	62.91	60.03
4	66.36	67.17	63.97	64.66	64.9	64.02	41.34	66.36	**80.29**	56.3
5	56.56	57.02	55.02	57.53	57.65	56.16	37.33	**62.27**	62.04	50.35
6	59.84	59.29	56.52	60.07	60.73	58.57	37.88	62.73	**78.31**	54.85
7	60.16	61.83	58.87	60.32	59.57	60.81	41.67	62.63	**68.92**	58.3
8	55.55	51.93	55.65	54.26	54.96	55.45	36.17	45.54	**63.43**	51.6

**Sequence-to-Sequence is the best model for few-shot learning:** While few-shot learning had a varying effect on models depending on the task, Seq2Seq performed best overall in all datasets.

**Low sample size causes high dialogue state tracking errors:** Because models have less training data for learning how to respond correctly to the user utterances, the dialogue state tracking errors increased in most of the models, which caused an increase in the error for B7 phrases (as shown in Supplementary Table S13 in SM 4). Moreover, most models perform worse in *personal(ised)* and *order details* phrases in task 8, however, there is no clear pattern for PB0 and PB1.

### 3.6.2 10,000 Dialogues

Table 8 and Table 9 show the per-response accuracy of the models on the Barista and Personalised Barista Datasets with 10,000 dialogues, respectively. The results for the Personalised Barista with Preferences Information Dataset and the error analysis based on phrase types are presented in the Supplementary Material section.

**TABLE 8:** The *test* set results of the Barista Dataset with 10,000 dialogues. The best performing methods (or methods within 0.1% of best performing) are given in bold for the per-response accuracy metric. The results show that on average and for task 8 (containing all tasks), Seq2Seq is the best performing model.

Task	MemN2N			Key-value	Seq2Seq	Supervised
	Hop1	Hop2	Hop3			
1	**100**	**100**	**100**	**100**	**99.9**	**100**
2	**100**	**100**	**99.99**	75.19	**100**	66.33
3	99.02	99.49	99.31	67.24	**100**	63.08
4	**100**	**99.99**	**99.99**	75.45	**100**	72.73
5	98.87	99.13	98.74	63.83	**99.93**	70.34
6	**100**	**99.99**	**100**	85.39	**99.98**	97.23
7	99.26	99.38	99.12	74.84	**99.98**	87.37

**TABLE 9:** The *test* set results of the Personalised Barista Dataset with 10,000 dialogues. The best performing methods (or methods within 0.1% of best performing) are given in bold for the per-response accuracy metric. The results show that on average, Split Memory is the best performing model, however, End-to-End Memory Network (MemN2N) is the best model for task 8 (containing all tasks).

Task	MemN2N			Split memory			Key-value	Profile	Seq2Seq	Supervised
	Hop1	Hop2	Hop3	Hop1	Hop2	Hop3				
0	35.19	35.79	37.01	39.1	40.8	40.82	34.3	34.53	34.89	**51**
1	68.17	67.44	68.19	69.73	70.57	69.54	**72.87**	63.54	64.74	68.17
2	69.39	69.37	69.65	**70.33**	70.21	69.63	68.83	64.15	65.07	63.44
3	69.78	71.22	70.8	69.5	71.56	**71.79**	47	58.09	65.27	61.59
4	80.05	79.91	79.42	**80.49**	79.88	79.23	47.77	62.48	75.29	61.1
5	69.44	**72.35**	72.05	69.28	70.88	71.6	42.72	57.1	58.47	58.09
6	**80.61**	79.73	79.12	79.42	79.43	79.01	44.62	32.92	75.03	58.55
7	78.05	77.7	76.9	78.05	**78.34**	78.19	41.79	73.03	75.78	61.5
8	**78.13**	77.79	**78.18**	77.32	77.41	77.32	39.88	55.35	71.34	56.42

**Sequence-to-Sequence is the best model for generic task-oriented dialogue:** Seq2Seq performs best in all models, achieving perfect or near-perfect accuracy in all tasks. MemN2N is also able to respond fully accurately in four out of seven tasks, which shows that more data improved its accuracy efficiently. The consistent results in both 1,000 and 10,000 dialogue sets confirm that these two models are suitable for generic task-oriented dialogue.

**None of the models is suitable for personalised task-oriented dialogue in real-world interactions:** While the accuracy of some of the models increased with more data, none of the models performed sufficiently well (i.e., above 90% accuracy) in the overall dialogue task (PB8) to be deployed in personalised long-term real-world interactions. As previously discussed, the underlying reason is their lack of ability to learn or use new customer names, as evidenced by the poor performance in task 0 in both Personalised Barista Datasets and the high percentage of error (i.e., mostly above 90%) in *personal(ised)* and *order details* phrases. On the other hand, Split Memory performed more accurately in both Personalised Barista Datasets with a higher number of samples, outperforming the previously best performing model, MemN2N, in most of the tasks except tasks 5, 6 and 8.

**High sample size improves model accuracy for generic task-oriented dialogue:** The performance of the models in the Barista Dataset improved with a higher number of *training* samples, as well as in the B7 and *other* phrases for the Personalised Barista Datasets. However, the percentage of error in *personal(ised)* and *order details* phrases varies between tasks, showing that the sample size has a varying effect on recalling or using customers’ names or preferences within the dialogue. Nevertheless, most models performed better on task 8 with more training data.

### 3.7 Training and Execution Times

In order to deploy a fully autonomous robot in a real-world interaction, its dialogue model should be able to respond within real-time, as it would highly affect the quality of the interaction. Table 1 (in Section 2.6) presents the training and test times for task 8 of the Personalised Barista Dataset^14^. The test time per example is calculated by dividing the overall execution time for the task (in the *test* set) by the number of utterances in each dataset (as given in Supplementary Table S2 in SM 1). Hence, the test time represents the average time the model takes to respond after the user utterance, which can be used to determine the real-time interaction capabilities.

**All models are suitable for real-time interaction:** MemN2N and Split Memory require the shortest time to train and respond to queries, hence, they are the most suitable for real-time interaction. Nevertheless, most models (i.e., all models except Supervised Embeddings) can respond under 1 s, which is sufficient for HRI. However, in verbal interaction with spoken dialogue systems, such as a robot, the time to process the audio (i.e., voice detection and automatic speech recognition) can increase the time to respond, thus, the interaction quality would be higher with a lower response time.

**End-to-End Memory Network and Split Memory are suitable to learn progressively:** Based on the short training and test times required for these models in all dataset sizes, new customer names and preferences can be learned progressively and incrementally from sequential interactions by re-training the models when a new entity or order is encountered, which can improve their task performance for personalised task-oriented dialogue.

## 4 Discussion

Although our evaluations show promise for generic task-oriented dialogue, the models did not achieve sufficient performance for user-specific personalisation in long-term interactions. Our results strongly suggest that state-of-the-art data-driven models currently lack lifelong learning capabilities.

One solution could be to re-train End-to-End Memory Network or Split Memory architectures, which were the best models for personalised dialogue, after encountering a new customer name or order since they can be trained in a short amount of time and have fast response times for real-time interactions. Training times for these models can be further reduced through a sparse read and write scheme (Rae et al., 2016). While re-training the models could also result in catastrophic forgetting, Memory Network models can be improved with methods to improve retrieval, such as word-based hashing (Weston et al., 2015; Dodge et al., 2016), clustering word embeddings (Weston et al., 2015), organising memories in a hierarchical system (e.g., Maximum Inner Search Product by Chandar et al. (2016), using match-type entities (Bordes et al., 2017) (especially for out-of-vocabulary entities) to help access relevant memories efficiently. Moreover, similar to the Split Memory architecture, an Episodic Memory Network could be developed by combining a generic pre-trained memory for responding to new users, with a user-specific memory that learns progressively through re-training or online learning for personalisation. Moreover, using previous interactions with other users (e.g., similar attributes between users or average model of all interactions) can help improve incomplete user profiles (Pei et al., 2021) and improve interactions for new users (Bak and Oh, 2019; Irfan et al., 2020b). For such an application, a dynamic weight predictor could help determine which part of the memory (generic or personal) should be given more weight to, similar to the work by Zheng et al. (2020b). The episodic memory could improve both lifelong and few-shot learning, because pre-trained information will be available for generic dialogue and new information will be learned sequentially per user. Such a model could also improve preference recalling as the previous user history will be contained in a separate memory. A *forgetting* mechanism (e.g., similar to the work of Kirkpatrick et al., 2017; Nugaliyadde et al., 2019; Wang et al., 2020a; Rae et al., 2020) can be introduced to remove or compress old memories for increasing the efficiency of memory retrieval and reducing catastrophic forgetting.

Another solution could be to combine a data-driven model with another approach to compensate for the deficiencies in the models, such as combining a generative model (e.g., Sequence-to-Sequence) with a Memory Network (Madotto et al., 2018; Zhang B. et al., 2020) or with transformers (Vaswani et al., 2017), such as in the work of Roller et al. (2020), Generative Pre-trained Transformer (GPT) (Radford et al., 2018, 2019; Brown et al., 2020; Zhang Y. et al., 2020), Bidirectional Encoder Representations from Transformers (BERT) (Devlin et al., 2019; Song et al., 2021), and Poly-encoders (Humeau et al., 2020; Li et al., 2020). Data-driven models can also be combined with graphical models (Zhou et al., 2020; Song et al., 2019; Moon et al., 2019; Shi et al., 2020; Wu B. et al., 2020; Xu et al., 2020), rule-based or slot-filling systems (Tammewar et al., 2018; Zhang Z. et al., 2019), a knowledge-base (Ganhotra and Polymenakos, 2018; Ghazvininejad et al., 2018; Luo et al., 2019; Yavuz et al., 2019; Moon et al., 2019; Wu et al., 2019; Lian et al., 2019; Zhang B. et al., 2020; Majumder et al., 2020; Tuan et al., 2021) or with automatic extraction of attributes from dialogue (Tigunova et al., 2019, 2020; Wu C.-S. et al., 2020, 2021; Ma et al., 2021) to improve the personalised entity selection in responses. Methods that adopt transfer learning (Genevay and Laroche, 2016; Lopez-Paz and Ranzato, 2017; Mo et al., 2017, 2018; Yang et al., 2017, 2018; Wolf et al., 2019; Golovanov et al., 2020), meta-learning (Finn et al., 2017; Santoro et al., 2016; Vinyals et al., 2016; Munkhdalai and Yu, 2017; Madotto et al., 2019; Zhang W.-N. et al., 2019; Song et al., 2020; Tian et al., 2021) and key-value memory structures (Xu et al., 2017; Kaiser et al., 2017; Zhu and Yang, 2018, 2020; de Masson d’Autume et al., 2019) could provide effective insights to alleviate data scarcity and enable quick adaption to various users through improving few-shot and lifelong learning capabilities of the dialogue models (Wang et al., 2020b).

Another line of attack could be to learn from users during deployment (similar to Zhao and Eskenazi, 2016; Hancock et al., 2019; Sreedhar et al., 2020; Liu, 2020; Irfan et al., 2020b), where the feedback could be the user response or emotions of the user to evaluate the user satisfaction with the agent’s responses. Adapting to the emotions of the user could improve the naturalness of the agent, and provide an additional level of personalisation within therapy, education or entertainment (Castellano et al., 2010; Irfan et al., 2020b; Churamani et al., 2020).

Future efforts could investigate how models with such further advancements perform on the Barista Datasets to determine which are suitable for responding accurately to users in personalised and non-personalised settings. However, in contrast to the typical chatbots, robots have multi-modal capabilities, such as vision and speech, which support more flexible and natural interaction, and increase their acceptability by users (Di Nuovo et al., 2018). While this work targeted possible errors and ways to overcome these for one of those modalities, namely for user recognition, another aspect that could highly affect the quality of the interaction and the performance of the dialogue architectures is speech recognition, as evidenced in the barista robot study. To evaluate how data-driven architectures perform with errors in speech recognition, one approach is to apply text-to-speech on the Barista Datasets, and add ambient and synthetic noise, followed by automatic speech recognition, similar to the proposed pipeline by Fang et al., 2020. However, this would not sufficiently model a real-world interaction with a robot due to the interference of the robot’s internal sounds (caused by motors) that may arise from body parts (e.g., for gestures) and head (e.g., for face tracking). Other problems that cannot be modelled with such a pipeline include latency due to connection and voice activity detection, quietly speaking users, user’s distance from the robot, foreign accents of non-native speakers (Irfan et al., 2020a) and speaker diarisation (van Waterschoot and Theune, 2021). An alternative approach is using partial Wizard-of-Oz (where the wizard responds to the users, but speech recognition is used for input) with a robot for data collection rather than a fully autonomous interaction where several repetitions and abrupt end of conversations could be encountered as observed in the barista robot study (Irfan et al., 2020a), which can make the data unusable for evaluations. Following these steps could help bring personalised robots, such as barista (Irfan et al., 2020a; de Berardinis et al., 2020) and bartender (Rossi and Rossi, 2021) robots within task-oriented domains, ready for real-world interaction through data-driven architectures.

## 5 Conclusion

This work highlights the lack of research on data-driven dialogue models for user-specific personalisation in long-term interactions, thereby, bridging the gap between neural conversational agents designed for short-term interactions and long-term human-robot interaction. The text-based Barista and Personalised Barista Datasets were created as testbeds for this purpose, consisting of simulated generic (i.e., non-personalised) and personalised task-oriented dialogues for long-term interactions between a barista and a customer, with the addition of incorrect user recognition and preference recalls for handling such situations in the real world human-robot interaction. The best performing models from other applications of personalisation, such as having a personality for open-domain dialogue and personalising based on generic user attributes in short-term task-oriented dialogue, were used for the evaluations on these datasets, namely, Supervised Embeddings, Sequence-to-Sequence, End-to-End Memory Network, Split Memory Network, Key-Value Profile Memory Network and Generative Profile Memory Network. The main conclusions from this work are: 1) Sequence-to-Sequence and End-to-End Memory Network are suitable for generic task-oriented dialogue, achieving up to near-perfect accuracy, 2) none of the models could reach 90% accuracy for personalised long-term dialogue, even when trained on a high number of (10,000) dialogue samples and user preferences information was provided, 3) underlying reason behind the inaccuracies of the models in the personalised task-oriented dialogue were identified to be the lack of capability to use new customer names or order items, the poor performance in recalling the user preferences, and user recognition errors, and 4) all models are suitable for real-time interactions in terms of response times. These results indicate that data-driven architectures are not yet ready to be deployed for personalised long-term human-robot interactions in the real world. However, we believe that the Barista Datasets and the key findings and suggestions from this work can be used as starting points to develop and test data-driven models for personalising long-term human-robot interactions.

## Data Availability

The datasets presented in this study can be found in online repositories. The names of the repository/repositories and accession number(s) can be found below: https://github.com/birfan/BaristaDatasets.

## Footnotes

1
https://github.com/birfan/BaristaDatasets.

2
https://parl.ai/.

3
https://github.com/google-research-datasets/Taskmaster.

4 Note that it is not clear how the users are identified in this dataset or whether the evaluation is sequential per user.

5
https://www.starbucks.com/menu.

6 Adapted Pepper is a Pepper robot (SoftBank Robotics Europe) with an improved microphone system (for lower noise) and a mounted camera (not used), which was created within the MuMMER project: http://mummer-project.eu.

7
http://doc.aldebaran.com/2-5/.

8
https://github.com/chaitjo/personalized-dialog.

9
https://parl.ai/.

10
www.docker.com.

11 Available online at: https://github.com/birfan/BaristaDatasets.

12 No statistical analysis was performed on the results, because each method could only be trained and tested once due to the lengthy training times and the available resources. Thus, the method of Bordes et al., 2017 is applied, which is a common practice in comparing performances of dialogue models in machine learning.

13 The *OOV* results of Joshi et al. (2017) are presented in: https://github.com/chaitjo/personalized-dialog/tree/master/MemN2N-split-memory.

14 The results are similar in other tasks.
