# Supplementary material for: Coffee With a Hint of Data: Towards Using Data-Driven Approaches in Personalised Long-Term Interactions
Source: Front Robot AI. 2021 Sep 28;8:676814. doi: 10.3389/frobt.2021.676814 (PMC8505524; doi:10.3389/frobt.2021.676814)
Supplement: Supplementary file 1 [file DataSheet1.pdf]

Supplementary Material

1 BARISTA DATASETS INFORMATION

Table S1. The percentage of *personal(ised)* (i.e., containing user name), *order details* (i.e., containing an item from the order) and *other (remaining)* phrase types in the bot utterances for the tasks of 1,000 and 10,000 dialogue Barista *test* set. The *candidate set* contains the unique utterances of the bot in all tasks and sets, and contains 4,149 utterances in 1,000 dialogues, and 5,207 in 10,000 dialogues.

Dataset Size	Phrases	B1	B2	B3	B4	B5	B6	B7
	Task Size	4,000	4,000	4,757	4,000	4,760	7,000	7,784
1,000	Personal(ised) (%)	25	0	0	0	0	14.29	12.85
	Order (%)	0	25	36.94	25	36.97	14.29	22.92
	Other (%)	75	75	63.06	75	63.03	71.43	64.23
	Vocabulary Size	432	331	350	333	351	446	463
	Task Size	40,000	40,000	47,467	40,000	47,506	70,000	77,490
10,000	Personal(ised) (%)	25	0	0	0	0	14.29	12.9
	Order (%)	0	25	36.8	25	36.85	14.29	22.57
	Other (%)	75	75	63.2	75	63.15	71.43	64.52
	Vocabulary Size	432	331	350	333	351	446	463

Table S2. The percentage of *personal(ised)* (i.e., containing user name or preference), *order details*, *other (remaining)* and Barista Task 7 (B7) phrase types in the bot utterances for the tasks of Second Interaction, 1,000 and 10,000 dialogue Personalised Barista *test* set.

Dataset Size	Phrases	PB0	PB1	PB2	PB3	PB4	PB5	PB6	PB7	PB8
Second Interaction (200 - 400)	Task Size	1,064	1,298	1,386	1,580	1,715	1,752	1,803	1,860	2,018
	Personal(ised) (%)	28.2	39.6	41.7	35.63	29.85	38.18	32.22	30.7	33.15
	Order (%)	24.81	27.27	27.71	29.62	32.13	29.79	32.61	31.72	32.11
	Other (%)	56.39	49.61	47.98	48.1	50.38	46.23	48.64	49.03	47.13
	B7 (%)	90.6	83.51	77.85	83.35	87.64	76.77	82.7	85.43	79.88
	Vocabulary Size	959	948	959	972	957	973	966	972	980
1,000	Task Size	3,471	3,489	3,768	4,728	5,141	5,125	5,460	5,873	6,233
	Personal(ised) (%)	54.74	54.46	55.94	45.79	36.96	46.89	38.39	36.69	37.98
	Order (%)	30.86	31.21	31.05	32.42	36.12	32.64	36.03	35.28	35.54
	Other (%)	40.33	40.13	39.49	40.82	44.43	40.1	43.77	43.35	42.44
	B7 (%)	74.07	74.2	67.94	75.36	82.49	70.54	78.21	80.33	76.54
	Vocabulary Size	959	959	971	975	965	983	977	981	989
10,000	Task Size	30,481	30,484	33,404	44,762	49,364	47,515	52,205	57,420	60,689
	Personal(ised) (%)	65.29	65.28	65.45	51.18	40.31	52.17	42.1	39.79	41.13
	Order (%)	33.07	33.08	33.09	34.25	38.39	34.25	38.03	37.05	36.81
	Other (%)	34.12	34.12	34	36.69	41.35	36.4	40.8	40.4	40.06
	B7 (%)	67.52	67.52	61.56	71.16	79.94	66.88	75.07	77.62	73.67
	Vocabulary Size	959	959	971	975	965	983	977	981	989

2 DATA-DRIVEN ARCHITECTURES

This section describes the data-driven dialogue models and their performance in the previous literature in detail and presents the hyperparameters used in the experiments for the Barista Datasets.

2.1 Models

2.1.1 Supervised Embeddings

Word embedding models are strong baselines for predicting the response given the previous conversation in both open-domain and task-oriented dialogue (Dodge et al., 2016; Bordes et al., 2017; Al-Rfou et al., 2016; Li et al., 2021). One common approach in the literature (Bai et al., 2009; Dodge et al., 2016; Bordes et al., 2017; Joshi et al., 2017) scores the summed bags-of-embeddings of the candidate responses against the summed bags-of-embeddings of the previous conversation, referred to as Supervised Embeddings. This approach corresponds to a Memory Network with no attention over memory (Dodge et al., 2016) and a classical information retrieval model where the matching function is learnt (Bordes et al., 2017).

This work uses the implementation from Joshi et al. (2017)[1]. Due to the structure of this method (i.e., binary bag-of-embeddings of unique words), the order of the words within the input, such as the user utterance, bot response or conversation context, is not preserved as the output is an embedding and not a sentence. Moreover, repeating words would also be lost in the embedding. Thus, this model may not be suitable for dialogue with this implementation.

2.1.2 Sequence-to-Sequence

Sequence-to-Sequence (Seq2Seq) model (Sutskever et al., 2014) is a generative model that uses a long short-term memory (LSTM) (Hochreiter and Schmidhuber, 1997; Graves, 2013) to read the input sequence (i.e., as the encoder) for obtaining a fixed-dimensional vector representation, and another LSTM to extract the output sequence from that vector (i.e., as the decoder). The model was found to be a strong baseline in task-oriented and open-domain dialogue (Vinyals and Le, 2015; Sordoni et al., 2015; Li et al., 2016a,b; Zhang et al., 2018).

This work uses the implementation from ParlAI[2] that was used in the ConvAI2[3] challenge (Dinan et al., 2020) with the Persona-Chat dataset.

2.1.3 End-to-End Memory Network

Humans tend to focus on salient parts of information for recalling the key aspects of a memory, or for efficiently accomplishing tasks. Similarly, attention mechanisms in deep learning focus on particular elements of a task, e.g., to respond to queries, based on a non-uniform weighting of the input to optimise the learning and recall processes. Such mechanisms can allow efficient memory handling and recalls in personalisation for long-term human-robot interaction, given the expanding volume of data over time.

End-to-End Memory Network (Weston et al., 2015; Sukhbaatar et al., 2015) (MemN2N) is an attention-based model with a long-term memory, where the input (e.g., user query) is weighted with a memory component to find the most relevant previous information, referred to as the *supporting facts* (e.g., the previous user or bot utterance in dialogue history), for producing an output (e.g., response). Multiple *hops* (i.e., iterating an output with the initial input in multiple layers) enforce the network to increase its attention

[1] `https://github.com/chaitjo/personalized-dialog`

[2] `https://github.com/facebookresearch/ParlAI/tree/master/projects/convai2/baselines/seq2seq`

[3] `http://convai.io`

on the correct supporting facts. The dialogue example from the *recognition error* task of the Personalised Barista with Preferences Information (PBPI2, Table S16 in SM 6) shows the attention weights on the *conversation context* (i.e., dialogue history) in varying hops.

A Memory Network can discover simple linguistic patterns based on verbal forms, such as (X, took, Y) for “Alice took the teacup.”, hence, it can generalise the meaning of their instantiations for previously unseen vocabulary (Weston et al., 2015), which is an essential quality, e.g., for learning new names in personalised long-term interactions. Moreover, the relative time of the events is encoded into the memory to retain the to-and-fro relations between events.

In task-oriented dialogue (Bordes et al., 2017), MemN2N outperformed Supervised Embeddings and information retrieval approaches, such as the Term Frequency-Inverse Document Frequency (TF-IDF) and the Nearest Neighbor. Moreover, the vanilla model outperformed Supervised Embeddings on the Personalized bAbI dialog dataset (Joshi et al., 2017).

Similar to the Supervised Embeddings, this work uses the implementation[1] from Joshi et al. (2017), which is a retrieval-based approach for vanilla MemN2N.

2.1.4 Split Memory Network

The Split Memory (Joshi et al., 2017) architecture combines a MemN2N for conversation context with another MemN2N for the user profile attributes (i.e., gender, age, favourite food and dietary preference) to enforce attention on the user’s profile, which is important for personalising a dialogue (e.g., for recommending a restaurant the user may like based on their favourite food). The outputs from both MemN2Ns are summed element-wise to get the final response of the bot for each conversation turn. For multiple hops, each MemN2N separately processes the output in multiple layers, and then the resulting outputs are summed.

Split Memory outperformed Supervised Embeddings in the Personalized bAbI dialog dataset in all tasks, and outperformed MemN2N in recommending the correct restaurant and conducting a full dialogue. On the other hand, Split Memory performed worse for responding to user queries and when the user requested changes, such as requesting a different type of cuisine than the previously requested one, suggesting that a simpler MemN2N model is more suitable for tasks that do not require compositional reasoning over various entries in the memory. In contrast to MemN2N, multiple hops may perform worse when there is more than one aspect of the user’s profile, such as favourite food and dietary preference, or memory event to focus on (Joshi et al., 2017), as evident in a dialogue example from the *recognition error* task of the Personalised Barista with Preferences Information (PBPI2) presented in Table S17 in SM 6.

This work uses the implementation[1] from (Joshi et al., 2017), which is a retrieval-based model.

2.1.5 Key-Value Profile Memory Network

Key-Value Memory Network (Miller et al., 2016) is an extension of retrieval-based MemN2N by storing facts in key-value structured memory slots. The keys are used to lookup relevant memories to the input (e.g., sharing at least one word with the input), and the corresponding values are read by taking their weighted sum using the assigned probabilities. Using hops (through repeated key addressing and value reading) enables focusing on and retrieving more pertinent information in subsequent accesses. If the key and value are set to be the same for all memories, the model becomes equivalent to the vanilla MemN2N.

Key-Value Memory Network was applied to open-domain dialogue (Zhang et al., 2018) by using the *training* set as the keys, and the values as the next dialogue utterance (e.g., user response). Correspondingly,

the model had a memory of past dialogues that can be used to predict responses to the current conversation. User profiles (*persona*) that consisted of multiple sentences of textual description (e.g., "I have a computer science degree") were used to find the relevant lines to combine with the input (cosine similarity), which is used to predict the next utterance, by summing the weighted sum of profile sentences with the input query, and ranking the candidate set responses to determine most suitable bot response. Thus, the model was called Key-Value Profile Memory Network, here referred to as Key-Value for brevity. For multiple hops, the summed value is used to attend over the keys and output a weighted sum of values as before, which is then used to rank the candidate set to predict the next utterance.

Key-Value outperformed Seq2Seq and Generative Profile Memory Network in both the automated metric (i.e., the accuracy of the next dialogue utterance when choosing between the correct response and distractor responses, known as *hits@1*) and human evaluation (in terms of fluency, engagingness and consistency) (Zhang et al., 2018).

This work uses the implementation[4] that was used at the ConvAI2 challenge with the Persona-Chat dataset.

2.1.6 Generative Profile Memory Network

Generative Profile Memory Network (Zhang et al., 2018), here referred to as Profile Memory for brevity, extends the Seq2Seq model by encoding the profile entries as individual memory representations in a Memory Network. The decoder attends over both the encoded profile entries and the conversation context.

Profile Memory was shown to outperform Seq2Seq for automated metrics (hits@1 and perplexity) in the Persona-Chat dataset, however, it performed considerably worse than Key-Value in hits@1, achieving a score of 0.125 in comparison to 0.511 by Key-Value.

This work uses the implementation from ParlAI[5] that was used in (Zhang et al., 2018).

2.2 Hyperparameters

The hyperparameters for the data-driven architectures used in this work correspond to the hyperparameters from the original implementations unless otherwise noted in the text. In contrast to the original work, we used 100 epochs for training each baseline to ensure the equal comparison between models, except for Key-Value and Supervised Embeddings, which were only trained for 25/15 epochs due to the vast amount of time required to train them. However, the number of epochs or training time in the original work are either less than or equal to ours. For instance, Key-Value Memory Network was trained for 20 hours (in equivalence to our computational power) on the Persona-Chat dataset (Zhang et al., 2018), whereas, the training lasted between 17 to 40 **days per task** in the 10,000 dialogues datasets, despite having 20% of its task size. In addition, the batch size of the Supervised Embeddings was increased to 128 (was 32 in the original implementation) to decrease the training time, and a batch size of 1 was used for Seq2Seq and Generative Profile Memory Network on the *test* and *OOV* sets due to out-of-memory errors. Key-Value Memory Network, Generative Profile Memory Network and Seq2Seq model do not contain out-of-vocabulary words, similar to the original work. *Previous user-bot labels* refers to all the previous exchanges within the dialogue before the *query* (current user response).

[4] `https://github.com/facebookresearch/ParlAI/tree/master/projects/convai2/baselines/kvmemnn`

[5] Note that while this baseline was used in (Zhang et al., 2018) with the Persona-Chat dataset, it was deprecated from the ParlAI library on March 2019. Thus, the last available version before the deprecation is used: `https://github.com/facebookresearch/ParlAI/tree/6a76a555ea84b06e2914cdea4c56a46a5f495821/projects/personachat`

Similar to (Joshi et al., 2017), the user profile is treated as a turn in the dialogue for MemN2N. The embeddings and memory have a fixed size, hence, the beginning of the conversation context may be cut off. The answer array is returned as a one-hot encoding. The Adam optimiser is used for minimising the cross entropy loss.

Split Memory has the same embedding and memory structures as MemN2N. The Adam optimiser is used for minimising the cross entropy loss. The profile attributes are added as separate entries in the memory before the start of the dialogue. In the Personalised Barista Datasets, the user profile can be updated during the conversation, such as for registering a new user or due to a recognition error, which is in contrast to the Personalized bAbI dialog dataset, where the user profile is not changed during the conversation. Thus, we overwrite the *profile memory* (i.e., MemN2N containing the information for the user profile attributes) with the new profile information, when a change of identity information occurs in dialogue. In contrast to the implementation that uses the most recent utterance in the context memory (differently from the reported results in (Joshi et al., 2017)), the full dialogue history is used in this work to improve the dialogue accuracy (e.g., 66.95% accuracy with the context, whereas 64.78% without the full context in PB8 for 1,000 dialogues).

Similar to the Split Memory, the user profile attributes are provided separately for Key-Value Memory Network, and are overwritten if updated during the conversation. Similar to (Zhang et al., 2018), only 1-hop model is evaluated due to the vast amount of time required to train the model, arising from the large size of the (key-value) pairs. In contrast to (Zhang et al., 2018), the model is trained on the Barista Datasets instead of using the weights from another model. Similar to (Zhang et al., 2018), but in contrast to the other methods, only the last bot utterance and the corresponding last user response are used instead of the full conversation context. This was reported to perform better in the original implementation, and the results in the Barista Datasets are in line with this finding, providing up to 20% increase in accuracy (e.g., 19.2% using context, 40.09% with only the utterance pair in PB8). Note that other methods were not evaluated in this way, either because the structure was not implemented or a difference was not reported in the original work. End-to-end training is enabled with standard backpropagation through stochastic gradient descent (SGD).

Similar to the work in (Zhang et al., 2018), GloVe embeddings (Pennington et al., 2014) is used for Generative Profile Memory Network, and the model is trained with the Adam optimiser. However, in contrast to the original implementation, the conversation context (i.e., history of the user and bot utterances) is used for the model, because this was found to improve the accuracy in the majority of the tasks in the Barista Datasets (e.g., for 1,000 dialogues datasets, 57.82% accuracy with context, 55.22% without context for the PB8). While the original implementation uses the user response-correct bot responses pairs in *training* for the conversation context, the user response-*model's prediction* pairs are used in the *validation*, *test* and *OOV* sets, which also performed better than using the correct response on the Barista Datasets (e.g., 57.82% accuracy with the model response in conversation context, 51.93% with correct bot response in context for PB8). Hence, this method was used, which also allowed for a fair comparison of this baseline to its performance in the Persona-Chat dataset. Similar to Split Memory, the profile attributes are separate and overwritten if updated during the conversation.

In contrast to Zhang et al. (2018), randomly initialised embeddings are used for the Seq2Seq model in this work instead of GloVe (Pennington et al., 2014) word embeddings, due to achieving a higher per-response accuracy (e.g., achieving 60.58% accuracy in comparison to 41.75% in task 8 for 1,000 dialogues in the Personalised Barista Dataset). Moreover, conversation *context* (i.e., all previous user-bot labels) is used for Seq2Seq, in contrast to (Zhang et al., 2018) due to higher accuracy, similar to Generative Profile Memory

Network. Similar to (Zhang et al., 2018), the model is trained with negative log likelihood and the user profile is prepended to the input sequence (i.e., concatenated to the beginning of the input).

Supervised Embeddings model is trained with SGD using a margin ranking loss to ensure that the correct targets are ranked higher than any other targets (i.e., *negative candidates*). Similar to (Joshi et al., 2017), the user profile is treated as a turn in the dialogue.

GeForce GTX 1080 Ti or Tesla V100-SXM3-32GB was used as the graphics processing unit (GPU), depending on the availability on the server.

Table S3. Hyperparameters of the models used in the experiments for the Barista Datasets. These correspond to the parameters from the original implementations (Joshi et al., 2017; Zhang et al., 2018), unless otherwise noted in text.

**Hyper-parameter**	**MemN2N**	**Split Memory**	**Key-Value**	**Profile**	**Seq2Seq**	**Supervised**
Learning Rate	0.001	0.001	0.1	0.001	3	0.01
Embedding Size	20	20	1000	300	256	32
Negative Candidates	100	100	10	All	All	100
Optimiser	Adam	Adam	SGD	Adam	SGD	Adam
User Profile	Dialogue turn	Separate	Separate	Separate	Prepended	Dialogue turn
Hops	1-3	1-3	1	1	-	-
Batch Size	32	32	1	128/ 1 (for test)	64/ 1 (for test)	128
Training Epochs	100	100	25	100	100	25/ 15 (for 10,000 dialogues)
Resource	1 GPU+ 1 CPU	1 GPU+ 1 CPU	16-18 CPUs	1 GPU+ 1 CPU	1 GPU+ 1 CPU	1 GPU+ 1 CPU
Vocabulary	all sets	all sets	*training*, *validation*, *test*	*training*, *validation*, *test*	*training*, *validation*, *test*	all sets
Conversation Context	Previous user-bot labels (may be cut off)	Previous user-bot labels (may be cut off)	Last bot-user label	Previous user-bot labels (training)/ Previous user-predicted bot responses (validation and test)	Previous user-bot labels	Previous user-bot labels

3 OUT-OF-VOCABULARY SET RESULTS

3.1 1,000 Dialogues

Table S4. The *out-of-vocabulary (OOV)* set results of the Personalised Barista Dataset with 1,000 dialogues. The best performing methods (or methods within 0.1% of best performing) are given in bold for the per-response accuracy metric. The results show that on average and for task 8 (containing all tasks), Supervised Embeddings is the best performing model.

**Task**	**MemN2N**			**Split Memory**			**Key-Value**	**Profile**	**Seq2-Seq**	**Super-vised**
	Hop1	Hop2	Hop3	Hop1	Hop2	Hop3				
0	41.73	42.88	41	43.52	43.63	41.96	20.88	40.69	39.94	**55.16**
1	47.53	46.52	43.01	46.82	47.33	44.89	20.84	47.23	46.01	**53.14**
2	43.59	45.02	43.83	44.11	44.93	45.02	20.29	47.8	42.73	**52.76**
3	43.08	42.01	38.38	43.63	44.38	41.78	19.8	45.64	43.83	**55.73**
4	44.29	43.46	42.52	42.95	44.54	41.4	16.45	47.51	49.24	**57.72**
5	42.64	42.84	40.99	39.61	44.37	40.07	16.52	43.76	43.53	**53.26**
6	40.73	44.32	45.82	43.79	42.75	39.71	32.46	46.97	42.82	**54.6**
7	41.21	43.91	41.99	39.38	40.03	41.04	14.53	47.88	46.74	**55.72**
8	42.47	40.56	39.31	42.79	39.06	43.88	13.87	39.91	46.26	**53.82**

Table S5. The *out-of-vocabulary (OOV)* set results of the Personalised Barista with Preferences Information Dataset with 1,000 dialogues. The best performing methods (or methods within 0.1% of best performing) are given in bold for the per-response accuracy metric. The results show that on average and for task 8 (containing all tasks), Supervised Embeddings is the best performing model, similar to the Personalised Barista Dataset.

**Task**	**MemN2N**			**Split Memory**			**Key-Value**	**Profile**	**Seq2-Seq**	**Super-vised**
	Hop1	Hop2	Hop3	Hop1	Hop2	Hop3				
0	41.41	41.52	40.11	40.83	39.56	40.77	17.35	40.54	39.39	**53.71**
1	46.57	46.77	44.18	46.52	43.21	42.65	17.79	46.31	43.42	**51.11**
2	44.35	44.21	40.96	40.96	43.64	41.72	16.27	45.84	43.64	**56.59**
3	40.87	45.88	44.02	40.43	39.68	38.3	14.99	44.42	45.25	**54.9**
4	45.73	43.89	42.77	41.94	46.89	42.34	10.99	47.29	45.55	**57.93**
5	40.99	42.37	38.61	37.99	38.65	37.92	11.33	44.41	43.3	**54.12**
6	44.74	45.92	42.02	44.32	44.21	45.4	11.3	46.55	42.96	**55.22**
7	41.66	39.8	40.98	41.92	38.4	40.91	11.76	47.46	44.07	**55.64**
8	41.63	40.13	40.66	40.38	37.09	43.19	9.92	43.38	40.41	**53.19**

Table S6. Percentage of errors in dialogue state tracking (DST), *personal(ised)*, *order details*, other and Barista Task 7 (B7) phrase types for 1,000 dialogue *out-of-vocabulary (OOV)* sets. The best performing methods (or methods within 0.1%) are given in bold for the error in per-response accuracy metric, and the error percentages within the phrase types are given in parentheses.

Task	Error Type	MemN2N			Split Memory			Key-Value	Profile	Seq2-Seq	Super-vised
		Hop1	Hop2	Hop3	Hop1	Hop2	Hop3				
B7	DST	19.49	11.5	13.48	-	-	-	26.57	-	**0.8**	10.08
	Person-al(ised)	**10.04** (77.80)	10.41 (80.60)	11.19 (86.70)	-	-	-	11.55 (89.50)	-	11.95 (92.60)	12.43 (96.30)
	Order	22.51 (99.89)	22.30 (98.91)	22.54 (100.00)	-	-	-	**21.38** (94.85)	-	22.54 (100.00)	21.56 (95.65)
	Other	12.02 (18.62)	5.76 (8.92)	6.26 (9.70)	-	-	-	34.30 (53.14)	-	**0.54** (0.84)	4.34 (6.72)
PB0	DST	23.19	4.07	2.45	24.23	22.49	4.71	31.33	2.02	2.97	**1.76**
	Person-al(ised)	52.96 (96.52)	52.15 (95.05)	53.05 (96.68)	**50.27** (91.62)	51.05 (93.05)	52.18 (95.10)	54.35 (99.04)	53.57 (97.62)	54.87 (99.99)	53.51 (97.52)
	Order	30.67 (99.89)	30.70 (99.99)	30.70 (99.99)	30.70 (99.99)	30.70 (99.99)	30.70 (99.99)	**30.41** (99.05)	30.70 (99.99)	30.70 (99.99)	30.64 (99.80)
	Other	0.64 (1.57)	**0.26** (0.64)	1.24 (3.07)	0.90 (2.21)	0.61 (1.50)	1.16 (2.86)	20.33 (50.28)	1.04 (2.57)	0.49 (1.21)	0.92 (2.29)
	B7	5.31 (7.18)	**4.97** (6.71)	5.95 (8.04)	5.60 (7.57)	5.31 (7.18)	5.86 (7.92)	25.01 (33.79)	5.75 (7.76)	5.20 (7.02)	5.57 (7.53)
PB1	DST	17.9	8.9	7.22	26.33	18.96	6.76	34.37	**0.36**	1.27	6.3
	Person-al(ised)	43.67 (95.45)	43.92 (96.01)	44.23 (96.68)	**42.20** (92.23)	42.81 (93.57)	43.72 (95.57)	45.14 (98.68)	44.08 (96.34)	44.94 (98.23)	44.59 (97.46)
	Order	28.83 (99.98)	28.83 (99.98)	28.83 (99.98)	28.77 (99.81)	28.83 (99.98)	28.83 (99.98)	**28.27** (98.05)	28.83 (99.98)	28.83 (99.98)	28.83 (99.98)
	Other	0.31 (0.67)	1.07 (2.33)	4.27 (9.33)	2.54 (5.56)	1.37 (3.00)	2.90 (6.33)	25.72 (56.23)	**0.20** (0.44)	0.56 (1.22)	8.08 (17.67)
	B7	8.80 (11.04)	9.56 (12.00)	12.76 (16.02)	10.98 (13.79)	9.86 (12.38)	11.39 (14.30)	33.91 (42.57)	**8.69** (10.91)	9.05 (11.36)	16.57 (20.81)
PB8	DST	10.74	16.4	18.31	11.55	17.59	12.46	59.84	9.48	**1.78**	17.59
	Person-al(ised)	**32.61** (92.63)	33.18 (94.23)	33.30 (94.58)	32.83 (93.25)	33.46 (95.03)	33.30 (94.58)	34.52 (98.05)	33.68 (95.65)	32.90 (93.43)	34.68 (98.49)
	Order	**33.05** (98.31)	**32.96** (98.03)	33.08 (98.40)	**33.05** (98.31)	**33.05** (98.31)	33.11 (98.50)	33.18 (98.68)	33.33 (99.15)	33.55 (99.80)	33.15 (98.59)
	Other	5.35 (11.83)	6.79 (15.02)	7.86 (17.37)	4.85 (10.73)	7.92 (17.51)	3.26 (7.20)	31.96 (70.65)	6.85 (15.15)	**1.28** (2.84)	1.66 (3.67)
	B7	24.76 (31.38)	26.23 (33.24)	27.07 (34.31)	24.29 (30.78)	27.42 (34.75)	22.69 (28.76)	49.92 (63.27)	26.35 (33.40)	20.85 (26.42)	**20.72** (26.26)
PBPI8	DST	15.24	16.96	15.71	16.24	16.71	9.7	74.71	**7.67**	8.48	8.36
	Person-al(ised)	33.08 (93.96)	33.68 (95.65)	33.24 (94.40)	33.24 (94.40)	33.15 (94.14)	33.62 (95.47)	**31.46** (89.34)	33.99 (96.54)	33.21 (94.31)	34.05 (96.71)
	Order	32.71 (97.29)	32.14 (95.61)	32.93 (97.94)	32.55 (96.82)	32.93 (97.94)	32.83 (97.66)	**29.86** (88.81)	33.05 (98.31)	33.02 (98.22)	32.68 (97.19)
	Other	5.98 (13.22)	7.48 (16.54)	6.60 (14.60)	7.23 (15.99)	10.30 (22.77)	3.76 (8.30)	39.03 (86.29)	**3.07** (6.78)	6.82 (15.09)	**3.16** (6.99)
	B7	25.16 (31.89)	26.10 (33.08)	25.76 (32.65)	26.23 (33.24)	29.70 (37.65)	23.07 (29.24)	57.06 (72.32)	22.44 (28.44)	26.38 (33.44)	**22.07** (27.97)

3.2 Second Interaction

Table S7. The *out-of-vocabulary (OOV)* set results of the Personalised Barista Dataset for Second Interaction set (*few-shot learning*). The best performing methods (or methods within 0.1% of best performing) are given in bold for the per-response accuracy metric. The results show that on average and for task 8 (containing all tasks), Supervised Embeddings is the best performing model, similar to the *out-of-vocabulary (OOV)* set of the Personalised Barista Dataset with 1,000 dialogues.

Task	Memory Networks			Split Memory			Key-Value	Profile	Seq2-Seq	Super-vised
	Hop1	Hop2	Hop3	Hop1	Hop2	Hop3				
0	**56.39**	56.02	54.42	54.51	51.69	55.17	18.98	56.02	55.45	41.35
1	56.38	53.38	55.25	53.75	**57.5**	51.31	23.83	50.47	56	50.66
2	52.62	54.52	46.65	48.73	54.16	53.44	20.98	**55.79**	55.33	52.87
3	54.38	50.58	51.24	53.31	50.74	54.05	21.49	55.12	55.54	**61.59**
4	53.54	56.03	55.71	55.14	52.25	55.47	16.4	56.51	53.78	**56.8**
5	46.58	48.21	46.42	48.68	47.2	46.5	15.47	49.92	54.43	**55.01**
6	47.66	50.91	51.81	48.11	47.96	51.13	18.5	53.25	53.47	**57.45**
7	51.47	51.17	52	53.43	53.43	49.43	16.3	53.66	50.42	**61.42**
8	45.51	50.56	48.18	50.42	50.63	50.28	15.64	44.04	52.24	**58.27**

Table S8. The *out-of-vocabulary (OOV)* set results of the Personalised Barista with Preferences Information Dataset for Second Interaction set (*few-shot learning*). The best performing methods (or methods within 0.1% of best performing) are given in bold for the per-response accuracy metric. The results show that on average, Sequence-to-Sequence is the best performing model, whereas Supervised Embeddings model performs best in task 8 (containing all tasks).

Task	Memory Networks			Split Memory			Key-Value	Profile	Seq2-Seq	Super-vised
	Hop1	Hop2	Hop3	Hop1	Hop2	Hop3				
0	53.38	55.73	53.95	**56.67**	55.36	55.55	26.5	56.3	55.64	25.95
1	**56.85**	**56.94**	54.03	53.66	56.29	55.25	22.61	51.22	54.97	42.97
2	51.9	54.79	50.81	49.01	53.35	53.35	23.24	**54.52**	**54.52**	47.98
3	53.39	51.98	50.66	48.02	54.38	47.52	21.65	50.58	53.06	**59.74**
4	53.46	54.82	53.7	53.22	**57.4**	50.4	22.03	54.66	56.59	57.25
5	49.22	49.38	47.9	44.09	49.61	43.23	16.41	47.59	**52.72**	51.19
6	51.06	50	48.19	46.9	46.07	50.68	15.03	51.89	**53.32**	44.93
7	51.17	53.58	50.64	50.79	48.91	50.26	19.09	52.53	55.47	**59.2**
8	48.53	45.72	48.95	49.72	46.21	48.25	18.02	52.17	52.66	**53.31**

3.3 10,000 Dialogues

Table S9. The *out-of-vocabulary (OOV)* set results of the Barista Dataset with 10,000 dialogues. The best performing methods (or methods within 0.1% of best performing) are given in bold for the per-response accuracy metric. The results show that on average and for task 8 (containing all tasks), Sequence-to-Sequence (Seq2Seq) is the best performing model.

Task	Memory Networks			Key-Value	Seq2Seq	Supervised
	Hop1	Hop2	Hop3			
1	**80.02**	79.73	76.14	75.26	78.53	78.46
2	72.39	50.5	67.58	18.65	**75**	64.73
3	52.14	49.65	53.7	7.97	61.99	**62.56**
4	49.64	71.11	66.79	10.99	**75**	73.81
5	61.13	53.01	41.9	5.94	**63.11**	46.68
6	57.64	65.69	58.29	28.21	**70.13**	65.65
7	56.62	49.45	59.2	31.25	**65.3**	65.04

Table S10. The *out-of-vocabulary (OOV)* set results of the Personalised Barista Dataset with 10,000 dialogues. The best performing methods (or methods within 0.1% of best performing) are given in bold for the per-response accuracy metric. The results show that in all tasks, Supervised Embeddings is the best performing model.

Task	Memory Networks			Split Memory			Key-Value	Profile	Seq2-Seq	Super-vised
	Hop1	Hop2	Hop3	Hop1	Hop2	Hop3				
0	36.42	36.2	36.4	35.13	38.59	39.08	30.61	35	35.38	**50.85**
1	38.13	37.25	37.84	41.58	41.34	38.74	29.41	35.42	35.99	**51.21**
2	40.73	40.9	39.71	33.48	39.04	40.08	28.3	38.28	36.38	**49.2**
3	31.27	32.58	30.72	38.9	29.66	31.88	18.6	39.02	37.76	**50.07**
4	32.4	35.1	37.27	41.63	32.19	32.52	15.47	43.33	42.76	**51.19**
5	33.54	33.14	32.08	32.2	34.91	34.34	19.58	39.91	38.79	**46.4**
6	35.55	40.63	32.71	29.48	28.28	39.27	18.48	18.75	43.13	**49.39**
7	35.65	26.58	30.47	29.54	35.97	37.46	15.5	39.35	38.27	**45.89**
8	32.3	32.56	35.97	34.36	31.81	34.0	15.48	37.28	41.64	**47.96**

Table S11. The *out-of-vocabulary (OOV)* set results of the Personalised Barista with Preferences Information Dataset with 10,000 dialogues. The best performing methods (or methods within 0.1% of best performing) are given in bold for the per-response accuracy metric. The results show that in all tasks, Supervised Embeddings is the best performing model.

Task	Memory Networks			Split Memory			Key-Value	Profile	Seq2-Seq	Super-vised
	Hop1	Hop2	Hop3	Hop1	Hop2	Hop3				
0	36.4	35.39	34.6	36.75	36.74	36.23	15.1	34.12	34.9	**50.45**
1	36.58	35.24	35.37	38.7	39.19	39.32	13.23	33.79	34.29	**51.28**
2	39.28	39.48	39.42	38.98	40.31	38.82	13.58	36.35	34.5	**50.24**
3	31.61	31.23	32.13	29.86	35.01	34.69	6.74	36.81	35.61	**50.4**
4	37.05	41.23	38.47	30.48	34.32	35.2	4.86	42.43	41.45	**51.29**
5	31.89	33.86	36.2	32.06	31.49	35.25	8.15	38.52	38.78	**48.28**
6	35.4	35.88	37.97	32.17	33.81	33.97	6.05	41.6	42.16	**51.54**
7	30.13	33.07	28.87	28.8	33.11	31.06	6.07	37.59	40.46	**41.31**
8	31.18	31.81	33.79	27.41	30.1	32.92	6.85	40.24	40.5	**48.23**

4 SECOND INTERACTION DATASET RESULTS

Table S12. The *test* set results of the Personalised Barista with Preferences Information Dataset with Second Interaction set (*few-shot learning*). The best performing methods (or methods within 0.1% of best performing) are given in bold for the per-response accuracy metric. The results show that on average and for task 8 (containing all tasks), Seq2Seq is the best performing model.

**Task**	**MemN2N**			**Split Memory**			**Key-Value**	**Profile**	**Seq2-Seq**	**Super-vised**
	Hop1	Hop2	Hop3	Hop1	Hop2	Hop3				
0	**59.87**	**59.96**	59.77	59.68	59.3	58.65	54.61	56.67	56.86	33.87
1	73.65	73.34	74.19	70.03	**74.5**	73.96	54.7	70.34	60.94	56.95
2	69.99	68.54	68.9	64.86	68.11	67.32	50.14	**72.01**	64.5	56.55
3	65.25	65.7	63.92	61.58	62.59	62.22	45.44	44.94	**71.33**	60.59
4	67.06	68.8	64.78	65.25	65.66	63.32	42.27	67.52	**78.66**	57.82
5	60.39	59.65	59.3	56.22	56.22	56.79	38.36	45.49	**74.43**	50.12
6	63.28	60.07	59.68	58.68	60.62	59.01	32.45	62.34	**76.82**	51.47
7	**64.14**	61.94	61.34	61.13	58.76	59.78	40.16	60.97	60.86	59.99
8	58.52	59.46	55.95	53.52	55.8	55.45	35.73	59.32	**74.43**	50.91

Table S13. Percentage of errors in dialogue state tracking (DST), *personal(ised)*, *order details*, other and Barista Task 7 (B7) phrase types for Second Interaction *test* sets. The best performing methods (or methods within 0.1%) are given in bold for the error in per-response accuracy metric, and the error percentages within the phrase types are given in parentheses.

**Task**	**Error Type**	**MemN2N** Hop1	Hop2	Hop3	**Split Memory** Hop1	Hop2	Hop3	**Key-Value**	**Profile**	**Seq2-Seq**	**Super-vised**
PB0	DST	7.8	5.45	2.91	11.09	4.32	3.29	19.27	1.5	**0.38**	16.64
	Person-al(ised)	27.07	27.54	27.07	26.41	26.41	27.07	**8.36**	28.20	28.20	28.01
		(95.98)	(97.65)	(95.98)	(93.65)	(93.65)	(95.98)	(29.66)	(99.98)	(99.98)	(99.32)
	Order	**22.46**	**22.37**	22.56	23.31	22.84	23.97	23.31	24.81	24.44	24.06
		(90.54)	(90.16)	(90.92)	(93.95)	(92.05)	(96.60)	(93.95)	(100.00)	(98.49)	(96.98)
	Other	0.56	0.94	0.94	0.66	0.66	0.38	21.33	0.38	**0.00**	29.14
		(1.00)	(1.67)	(1.67)	(1.17)	(1.17)	(0.67)	(37.83)	(0.67)	(0.00)	(51.67)
	B7	**13.63**	13.91	14.10	14.57	14.10	14.94	36.47	15.79	15.04	43.80
		(15.04)	(15.35)	(15.56)	(16.08)	(15.56)	(16.49)	(40.25)	(17.43)	(16.60)	(48.34)
PB1	DST	6.24	2.47	3.16	6.01	3.24	3.39	28.51	**0.15**	0.39	5.39
	Person-al(ised)	16.49	16.02	15.95	17.03	**14.79**	15.64	15.87	16.26	29.04	30.35
		(41.63)	(40.47)	(40.27)	(43.00)	(37.35)	(39.49)	(40.08)	(41.05)	(73.35)	(76.65)
	Order	**13.94**	14.41	15.02	15.02	14.56	14.79	25.73	15.56	21.65	23.96
		(51.14)	(52.83)	(55.09)	(55.09)	(53.40)	(54.24)	(94.36)	(57.07)	(79.39)	(87.86)
	Other	0.31	0.23	0.62	0.31	0.39	0.69	19.41	2.16	**0.00**	9.94
		(0.62)	(0.47)	(1.24)	(0.62)	(0.78)	(1.40)	(39.13)	(4.35)	(0.00)	(20.03)
	B7	9.32	9.71	10.71	9.71	9.94	10.55	29.51	12.48	**8.40**	19.57
		(11.16)	(11.62)	(12.82)	(11.62)	(11.90)	(12.64	(35.33)	(14.95)	(10.06)	(23.43)
PB8	DST	10.9	13.73	11.2	10.36	9.17	10.01	49.65	5.2	**1.73**	9.07
	Person-al(ised)	25.82	27.06	26.36	24.78	25.02	24.98	**19.52**	33.00	21.56	29.78
		(77.88)	(81.62)	(79.53)	(74.74)	(75.49)	(75.34)	(58.90)	(99.56)	(65.03)	(89.84)
	Order	**26.26**	26.91	25.62	28.64	28.74	27.45	30.97	32.06	27.21	30.43
		(81.79)	(83.80)	(79.79)	(89.20)	(89.51)	(85.50)	(96.45)	(99.85)	(84.72)	(94.76)
	Other	2.58	4.31	2.53	3.22	2.18	2.33	24.98	1.78	**0.15**	9.86
		(5.47)	(9.15)	(5.36)	(6.83)	(4.63)	(4.94)	(52.99)	(3.79)	(0.32)	(20.92)
	B7	17.29	19.72	17.34	19.82	19.18	18.53	42.47	20.91	**14.92**	27.55
		(21.65)	(24.69)	(21.71)	(24.81)	(24.01)	(23.20)	(53.16)	(26.18)	(18.67)	(34.49)
PBPI8	DST	12.09	12.49	16.85	10.6	9.51	11.11	59.27	**0.79**	1.68	13.28
	Person-al(ised)	23.44	23.49	25.97	26.36	26.16	25.22	**18.53**	21.01	20.91	29.19
		(70.71)	(70.86)	(78.33)	(79.53)	(78.93)	(76.09)	(55.91)	(63.38)	(63.08)	(88.05)
	Order	21.51	22.10	23.04	27.80	25.12	26.51	20.71	28.84	**12.49**	29.58
		(66.98)	(68.83)	(71.76)	(86.58)	(78.24)	(82.56)	(64.51)	(89.82)	(38.89)	(92.13)
	Other	3.77	3.07	3.72	2.82	2.97	2.38	26.16	**0.10**	0.30	10.70
		(7.99)	(6.52)	(7.89)	(5.99)	(6.31)	(5.05)	(55.52)	(0.21)	(0.63)	(22.71)
	B7	16.70	15.86	17.00	18.88	16.95	18.09	43.90	19.57	**4.41**	28.74
		(20.91)	(19.85)	(21.28)	(23.64)	(21.22)	(22.64)	(54.96)	(24.50)	(5.52)	(35.98)

5 10,000 DIALOGUES DATASET RESULTS

Table S14. The *test* set results of the Personalised Barista with Preferences Information Dataset with 10,000 dialogues. The best performing methods (or methods within 0.1% of best performing) are given in bold for the per-response accuracy metric. The results show that on average and for task 8 (containing all tasks), Split Memory is the best performing model.

Task	MemN2N			Split Memory			Key-Value	Profile	Seq2-Seq	Super-vised
	Hop1	Hop2	Hop3	Hop1	Hop2	Hop3				
0	34.39	34.56	35.59	37.94	36.69	38.63	**66.15**	33.78	34.37	50.43
1	67.66	67.46	67.73	69.37	67.84	69.99	69.26	62.24	63.82	**70.33**
2	68.06	69.24	68.76	68.31	69.69	68.89	**74.23**	64.44	63.13	67.54
3	75.15	75.05	75.39	75.75	75.38	**76.01**	54.47	58.88	72.05	66.3
4	79.58	79.75	79.21	77.3	**80.49**	79.65	61.12	62.28	76.51	66.66
5	75.45	**75.94**	75.61	75.38	74.91	75.18	55.24	57.51	70.72	63.12
6	**79.41**	**79.38**	79.25	74.53	72.03	75.84	56.67	61.29	74.9	61.58
7	80.73	79.56	80.34	72.27	80.79	**81.1**	49.79	55.63	71.8	66.39
8	**81.07**	79.39	80.85	72.82	63.38	**81.02**	46.64	58.45	75.93	62.92

Table S15. Percentage of errors in dialogue state tracking (DST), *personal(ised)*, *order details*, other and Barista Task 7 (B7) phrase types for 10,000 dialogue *test* sets. The best performing methods (or methods within 0.1%) are given in bold for the error in per-response accuracy metric, and the error percentages within the phrase types are given in parentheses.

Task	Error Type	MemN2N Hop1	MemN2N Hop2	MemN2N Hop3	Split Memory Hop1	Split Memory Hop2	Split Memory Hop3	Key-Value	Profile	Seq2-Seq	Super-vised
B7	DST	**0.02**	**0.01**	**0.02**	-	-	-	**0**	-	**0**	0.33
	Person-al(ised)	**0.01** (0.09)	**0.01** (0.07)	**0.01** (0.09)	-	-	-	**0.00** (0.00)	-	**0.00** (0.00)	1.06 (8.20)
	Order	0.73 (3.21)	0.60 (2.68)	0.86 (3.83)	-	-	-	22.24 (98.54)	-	**0.02** (0.08)	10.80 (47.86)
	Other	**0.01** (0.01)	**0.00** (0.01)	**0.00** (0.00)	-	-	-	2.92 (4.53)	-	**0.00** (0.00)	0.77 (1.19)
PB0	DST	21.99	3.22	0.79	27.97	26.36	23.58	33.16	1.36	0.78	**0.28**
	Person-al(ised)	64.39 (98.62)	63.72 (97.59)	62.05 (95.04)	60.41 (92.53)	58.71 (89.92)	**58.00** (88.84)	64.09 (98.16)	63.58 (97.38)	64.05 (98.10)	63.92 (97.90)
	Order	32.89 (99.46)	32.97 (99.71)	33.05 (99.94)	32.95 (99.63)	32.96 (99.66)	32.43 (98.05)	**32.09** (97.03)	33.07 (100.00)	33.07 (100.00)	33.05 (99.95)
	Other	**0.01** (0.02)	**0.00** (0.01)	**0.00** (0.00)	**0.01** (0.02)	**0.01** (0.02)	**0.01** (0.02)	1.02 (2.98)	1.30 (3.80)	0.47 (1.37)	0.74 (2.17)
	B7	**0.42** (0.62)	**0.50** (0.74)	0.57 (0.85)	**0.48** (0.71)	**0.49** (0.72)	0.56 (0.84)	1.60 (2.38)	1.89 (2.80)	1.06 (1.56)	1.32 (1.95)
PB1	DST	13.43	10.19	**0.13**	15.41	13.29	13.68	10.62	1.25	0.24	**0.18**
	Person-al(ised)	31.21 (47.80)	31.96 (48.96)	31.26 (47.89)	29.69 (45.48)	29.22 (44.76)	29.79 (45.63)	**25.27** (38.71)	34.85 (53.38)	34.27 (52.50)	41.44 (63.49)
	Order	16.53 (49.96)	16.66 (50.37)	16.61 (50.20)	16.62 (50.23)	16.62 (50.24)	16.58 (50.12)	**16.19** (48.95)	20.01 (60.50)	19.85 (60.02)	25.78 (77.94)
	Other	**0.02** (0.05)	**0.02** (0.05)	**0.02** (0.05)	**0.03** (0.09)	**0.03** (0.08)	**0.00** (0.00)	1.26 (3.70)	1.01 (2.96)	0.38 (1.11)	0.34 (1.01)
	B7	**0.50** (0.73)	**0.60** (0.89)	**0.55** (0.81)	**0.57** (0.85)	**0.57** (0.85)	**0.57** (0.85)	1.86 (2.76)	1.61 (2.39)	0.98 (1.45)	0.93 (1.38)
PB8	DST	6.89	5.64	2.19	**0.59**	8.79	1.01	50.18	**0.59**	**0.62**	3.66
	Person-al(ised)	20.98 (51.00)	21.21 (51.56)	**20.55** (49.97)	21.81 (53.02)	21.55 (52.40)	21.56 (52.43)	27.09 (65.87)	25.71 (62.50)	25.96 (63.13)	32.10 (78.04)
	Order	**12.87** (34.95)	12.99 (35.30)	13.10 (35.58)	**12.90** (35.04)	**12.95** (35.17)	12.99 (35.28)	34.06 (92.54)	33.40 (90.73)	17.59 (47.79)	32.74 (88.93)
	Other	**0.02** (0.04)	**0.02** (0.06)	**0.03** (0.09)	**0.03** (0.09)	**0.04** (0.09)	**0.08** (0.19)	14.20 (35.45)	0.31 (0.78)	**0.03** (0.08)	4.69 (11.70)
	B7	**0.88** (1.20)	0.98 (1.33)	1.21 (1.64)	**0.87** (1.19)	0.98 (1.33)	1.09 (1.47)	31.32 (42.52)	18.94 (25.71)	2.78 (3.78)	19.24 (26.12)
PBPI8	DST	**0.31**	0.48	0.58	5.84	4.67	4.67	43.87	**0.33**	**0.3**	3.46
	Person-al(ised)	**18.12** (44.05)	19.76 (48.04)	18.95 (46.06)	21.60 (52.52)	26.43 (64.26)	26.43 (64.26)	25.62 (62.30)	22.76 (55.34)	21.71 (52.79)	26.16 (63.61)
	Order	**9.81** (26.66)	**9.85** (26.76)	**9.89** (26.86)	16.17 (43.94)	25.30 (68.73)	25.30 (68.73)	28.18 (76.56)	29.97 (81.42)	12.46 (33.85)	27.39 (74.41)
	Other	**0.01** (0.02)	**0.03** (0.09)	**0.02** (0.06)	1.21 (3.03)	1.24 (3.08)	1.24 (3.08)	8.92 (22.27)	0.16 (0.40)	0.21 (0.53)	3.25 (8.11)
	B7	**0.81** (1.10)	**0.83** (1.13)	**0.90** (1.23)	5.58 (7.57)	10.18 (13.82)	10.18 (13.82)	26.06 (35.37)	18.79 (25.51)	2.36 (3.20)	17.62 (23.91)

6 DIALOGUE EXAMPLES

Table S16. A dialogue example from the *recognition error* task (2) of the Personalised Barista with Preferences Information Dataset (PBPI2) shows the attention weights in the End-to-End Memory Network (MemN2N) model for varying hops. Zero attention weight signifies a very small value ($< 10^{-5}$).

Time	Speaker	Conversation Context	Hop1	Hop2	Hop3
1		True , 182 , Tom Welling , large , latte	0.029	0	0
2	Customer	Hey there !	0.115	0.00004	0
3	Barista	Hello , Tom Welling ! Would you like to have a large latte again ?	0.047	0	0
4	Customer	That is not my name .	0.067	0.0002	0
5	Barista	I am terribly sorry ! May I take your name ? api_call getCustomerName	0.041	0.0002	0
6	Customer	It is Anne Hathaway .	0.334	0.04	0
7	Barista	Let me see if I have any previous records of you , Anne .	0.258	0.023	0
8		True , 23 , Anne Hathaway , small , mocha , blueberry muffin	0.108	0.937	1
**Customer Input**		Okay .			
**Correct Response**		I thought you looked familiar , **Anne** ! Would you like a **small mocha** and a **blueberry muffin** again ?			
**Predicted Response**		I thought you looked familiar , **Anne** ! Would you like a **small mocha** and a **blueberry muffin** again ?			

Table S17. A dialogue example from the *recognition error* task (2) of the Personalised Barista with Preferences Information Dataset (PBPI2) shows the attention weights in the Split Memory model for varying hops. Split Memory allows focusing attention separately on the user profile (i.e., the customer's identity and most preferred order), in addition to the last bot response (containing the customer's name to be used in the response), which reinforces dialogue state tracking and predicting the correct response. Preferences information helps choose the correct items in the suggestion, which decreases the risk of mixing customers (and preferences). Hops facilitate focusing attention to relevant inputs, however, it can decrease the performance when there are multiple target items (for preference suggestion or order confirmation), as evident in Hop 3. Zero attention weight signifies a very small value ($< 10^{-5}$).

		Profile	Hop1	Hop2	Hop3
		True	0.077	0.012	0.00002
		23	0.087	0.033	0.031
		Anne Hathaway	0.24	0.412	0.777
		small	0.087	0.004	0.00002
		mocha	0.244	0.297	0.18
		blueberry muffin	0.265	0.241	0.012
**Time**	**Speaker**	**Conversation Context**			
1	Customer	Hey there !	0.145	0	0
2	Barista	Hello , Tom Welling ! Would you like to have a large latte again ?	0.06	0	0
3	Customer	That is not my name .	0.151	0.0002	0
4	Barista	I am terribly sorry ! May I take your name ? api_call getCustomerName	0.055	0.006	0
5	Customer	It is Anne Hathaway .	0.251	0.00009	0
6	Barista	Let me see if I have any previous records of you , Anne .	0.339	0.994	1
**Customer Input**		Okay .			
**Correct Response**		I thought you looked familiar , **Anne** ! Would you like a **small mocha** and a **blueberry muffin** again ?			
**Predicted Response**		I thought you looked familiar , **Anne** ! Would you like a **small mocha** and a **blueberry muffin** again ?			

Customer: Hello!
Barista: Hello! Which drink can I get for you?
Customer: A **hot chocolate** please.
Barista: Which size?
Customer: **Small** cup.
Barista: Would you like anything else with that?
Customer: I would like a **doughnut** please.
Barista: So that would be a **small hot chocolate** and a **doughnut**. Is that right?
Customer: Erm, I would like a **slice of banana bread** actually.
Barista: Sure! So that would be a **small hot chocolate** and a **slice of banana bread**. Is that right?
Customer: Let's change slice of banana bread to a **croissant** instead.

Correct Response: *Sure! So that would be a **small hot chocolate** and a **croissant**. Is that right?*
Predicted Response: *Sure! So that would be a **small hot chocolate** and a **slice of banana bread**. Is that right?*

Figure S1. Incorrectly recalling the previous order item during a change of order in task 7 of the Barista Dataset within the Sequence-to-Sequence (Seq2Seq) model.

Customer: Hey there!
Barista: Hello! Which drink can I get for you?
Customer: Can I get a **cordusio**?
Barista: Which size?
Customer: **Large**, thank you!
Barista: Would you like anything else with that?
Customer: May I get a **classic oatmeal**?
Barista: So that would be a **large cordusio** and a **classic oatmeal**. Is that right?
Customer: Oh I am sorry, but I would like an **espresso** instead.

Correct Response: *Sure! So that would be a **large espresso** and a **classic oatmeal**. Is that right?*
Predicted Response: *Sure! So that would be a **large espresso**. Is that right?*

Figure S2. Missing parts of the order during change of order in task 7 of the Barista Dataset in the Sequence-to-Sequence (Seq2Seq) model.

Customer: Hey there!
Barista: Hello! Which drink can I get for you?
Customer: **Green tea**, please?
Barista: Which size?
Customer: **Large**, thank you!
Barista: Would you like anything else with that?
Customer: I would like a **chocolate caramel shortbread**.

Correct Response: *So that would be a* ***large green tea*** *and a* ***chocolate caramel shortbread****. Is that right?*
Predicted Response: *So that would be a* ***large green tea*** *and a* ***chocolate chip cookie****. Is that right?*

Figure S3. Dialogue example for incorrect item that does not occur in the conversation context in task 7 of the Barista Dataset in the End-to-End Memory Network (MemN2N) with 3 hops.

Profile: True, 96, Ariana Grande

Customer: Hi!

Correct Response: *Hello, Ariana Grande! Would you like to have a medium caramel macchiato again?*
Predicted Response: *Hello, Ariana?*

Figure S4. Grammatically incorrect bot response with missing words in task 8 of the Personalised Barista Dataset in the Profile Memory.

Profile: True, 157, Russell __UNK__

Customer: Hey there!

Correct Response: *Hello, Russell Brand! Would you like to have a* ***short peach iced tea*** *and a* ***chicken and pesto panini*** *again?*
Predicted Response: *Hello, Russell Brand! Would you like to have a* ***short raspberry lemonade*** *again?*

Figure S5. Key-Value can use out-of-vocabulary words, i.e., new customer names and order items (Brand, short, raspberry lemonade). However, the customer preference was incorrectly recalled. Russell is a first name in the *training* set. The __UNK__ is the special token used to represent words that are not in the vocabulary in ParlAI framework. Despite the special token in the profile and the conversation context, Key-Value is able to learn and use those new words.
